# Supplementary material for: Sorting polymerization in a bichannel metal-organic framework
Source: Nat Commun. 2025 Aug 5;16:6984. doi: 10.1038/s41467-025-62322-8 (PMC12325995; doi:10.1038/s41467-025-62322-8)
Supplement: Supplementary file 1 — Supplementary Information [file 41467_2025_62322_MOESM1_ESM.pdf]

Sorting polymerization in a bichannel metal-organic framework

Keat Beamsley, Nobuhiko Hosono*, Takashi Uemura*

Table of contents

1. Supplementary Figures ········ 2

2. Supplementary Tables ········ 30

3. Supplementary Methods ········ 32

4. Supplementary References ········ 36

1. Supplementary Figures

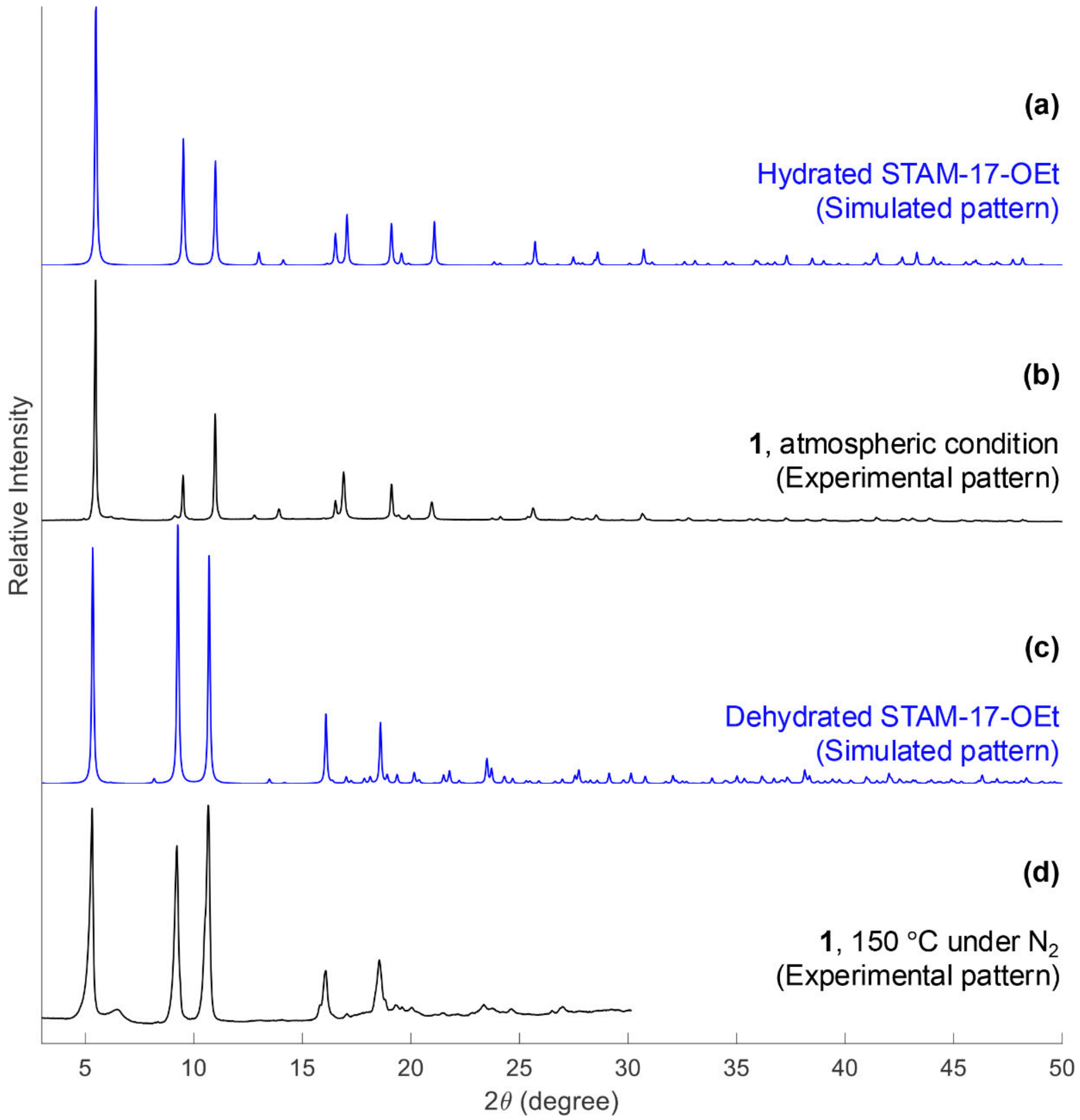

Supplementary Fig. 1. PXRD patterns of STAM-17-OEt (a, c) simulated from reported structural data[1] compared to the experimental patterns of **1** (b, d) measured in Bragg-Brentano (BB) reflection mode. We used STAM-17-OEt, which is one of several isoreticular MOFs with the same topology as STAM-1, as the reference since its single-crystal X-ray structural data in both its gate-open and gate-closed phases have been previously reported.[1] Clear correlation of the peaks is especially clear in the low-angle region. Similar patterns for both the hydrated and dehydrated forms strongly suggest these MOFs are isoreticular, thus the STAM-17-OEt[1] crystal structures were used as a starting point to develop gate-open and gate-closed models for **1** by density functional theory (DFT) calculations (Supplementary Method 3).

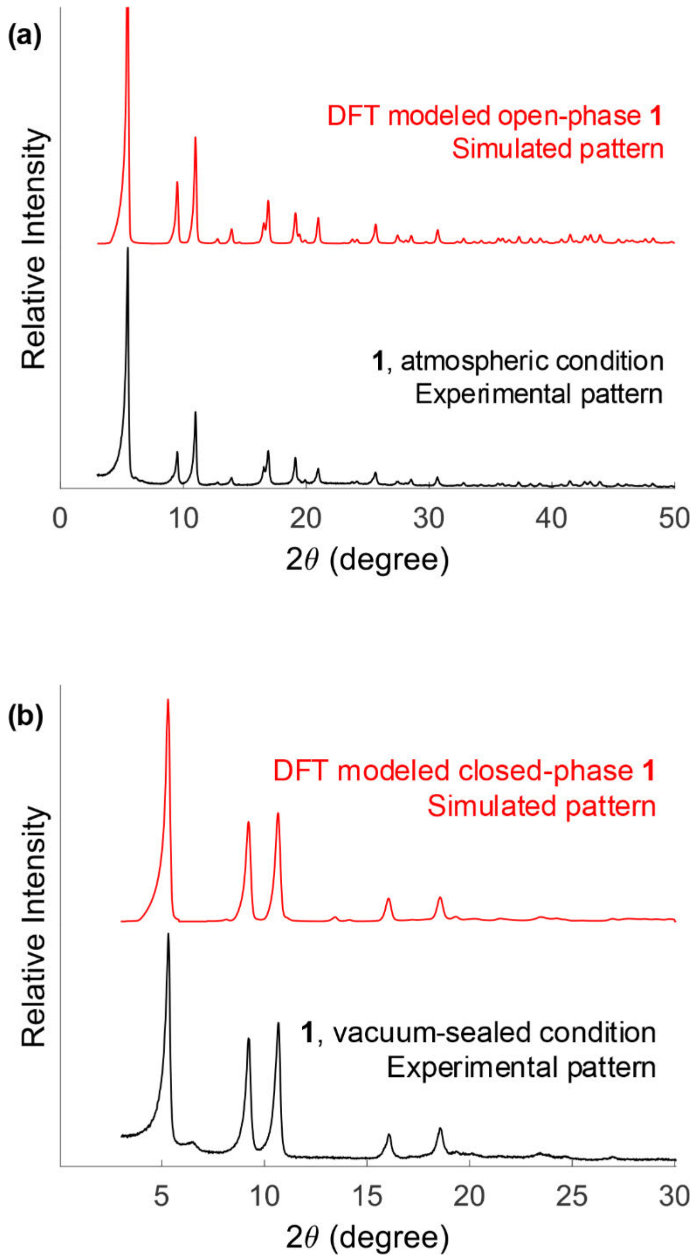

Supplementary Fig. 2. PXRD patterns of **1** measured by Debye-Scherrer method in (a) open-phase under atmospheric conditions, (b) closed-phase under vacuum following activation at 150 °C. Simulated patterns of the DFT-optimized models described in Supplementary Method 3 are provided for comparison using the same profile parameters as determined by Le Bail fitting described in Supplementary Method 2. Note that these simulations are presented without background correction, but backgrounds were indeed fitted during Le Bail analysis. Successful reproduction of the entire powder profile confirms these models are structurally accurate.

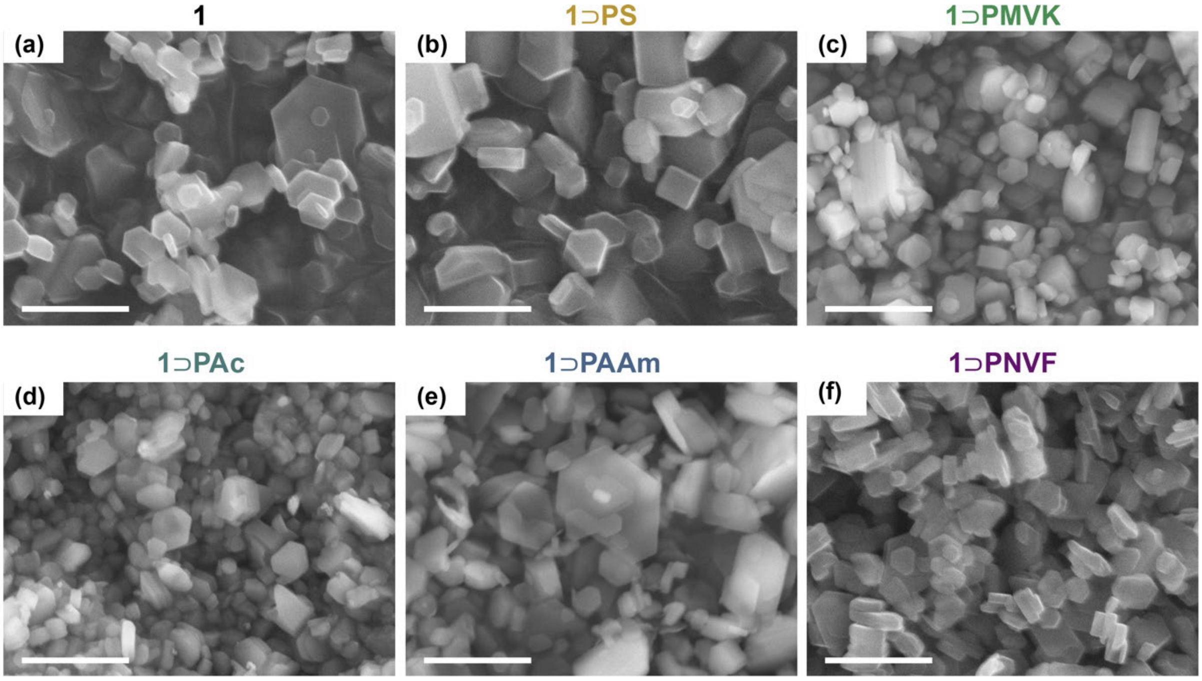

Supplementary Fig. 3. SEM images of (a) **1** and its composites with (b) **PS**, (c) **PMVK**, (d) **PAc**, (e) **PAAm**, and (f) **PNVF**. Scale bar: 1 μm. The same hexagonal morphology is observed in all cases. No amorphous material was observed outside the crystals, indicating polymerization occurred on the inside.

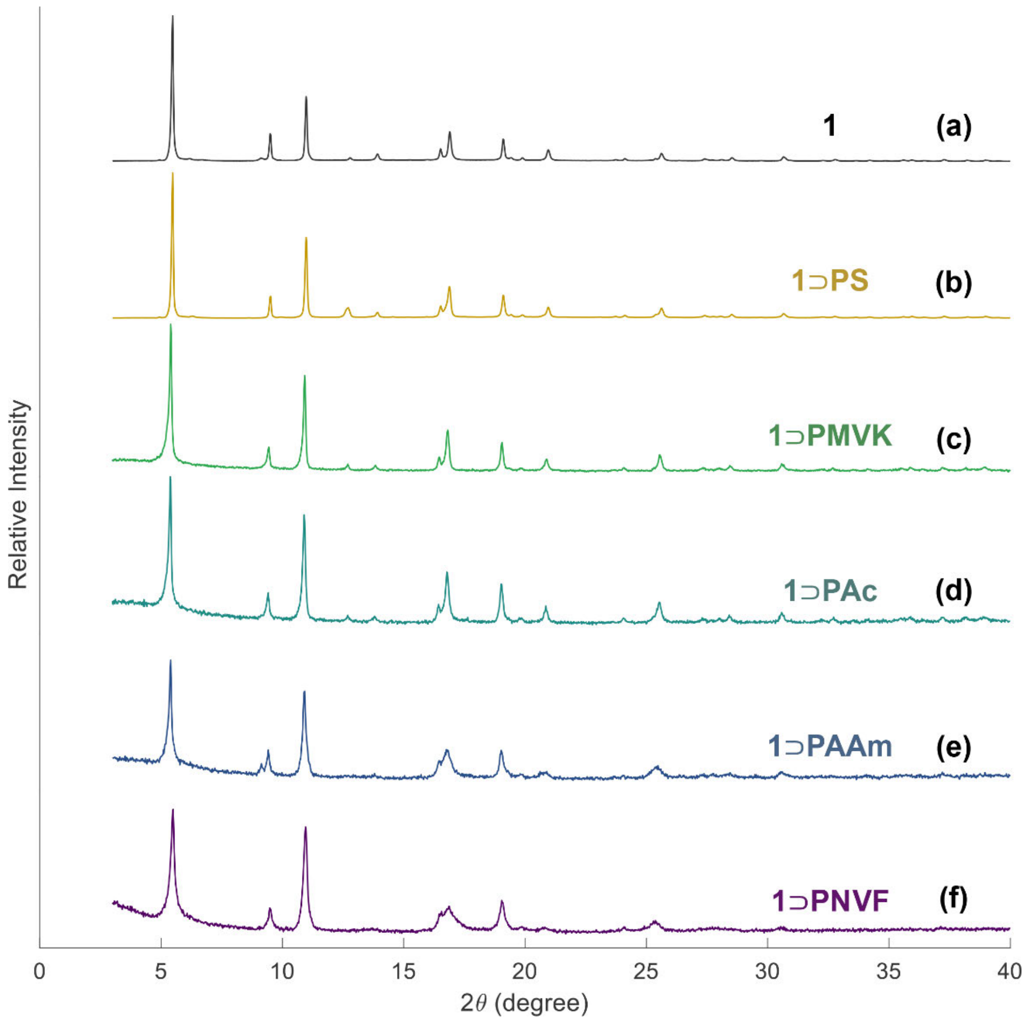

Supplementary Fig. 4. PXRD patterns of (a) **1** and its composites with (b) **PS**, (c) **PMVK**, (d) **PAc**, (e) **PAAm**, and (f) **PNVF**. As measurements were performed in Bragg-Brentano (BB) mode under atmospheric conditions, all PXRD patterns display the open phase here.

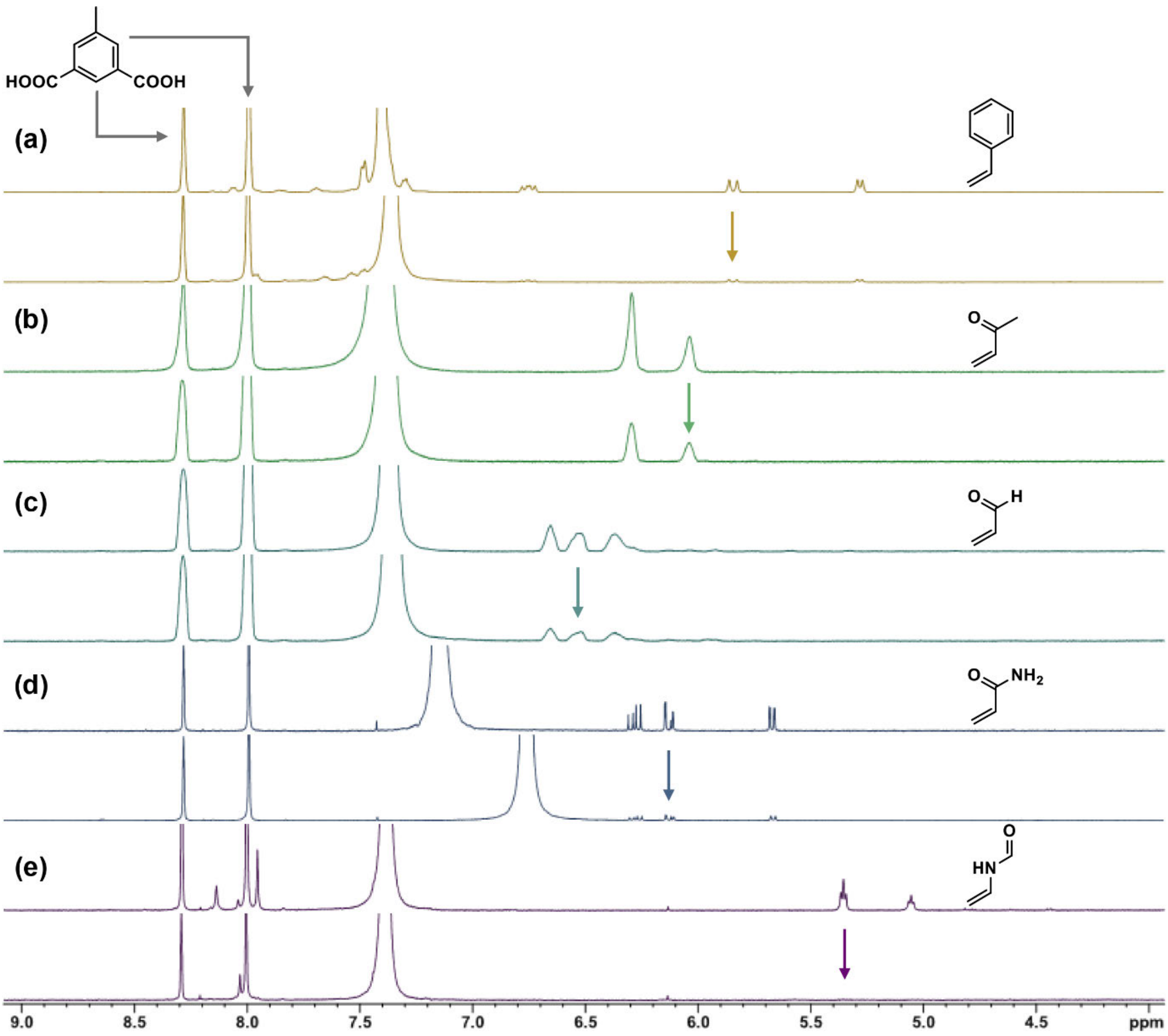

Supplementary Fig. 5. ^{1}H NMR spectra of DMSO-d_6/DCl (9/1, v/v)-digested composites of **1** with (a) **S**, (b) **MVK**, (c) **Ac**, (d) **AAm**, and (e) **NVF**. Two spectra are shown for each—Upper: before polymerization. Lower: after polymerization. The monomer loading ratio and conversion were calculated based on the integral values of monomer vinyl peaks in the 5.0-7.0 ppm region, normalized against mip peaks at 8.0 and 8.3 ppm. The decrease in monomer peaks after polymerization indicated monomer conversion to polymer. The large peak seen shifting between 6.5 and 7.5 ppm is attributed to the residual proton in DCl and shifts with concentration.

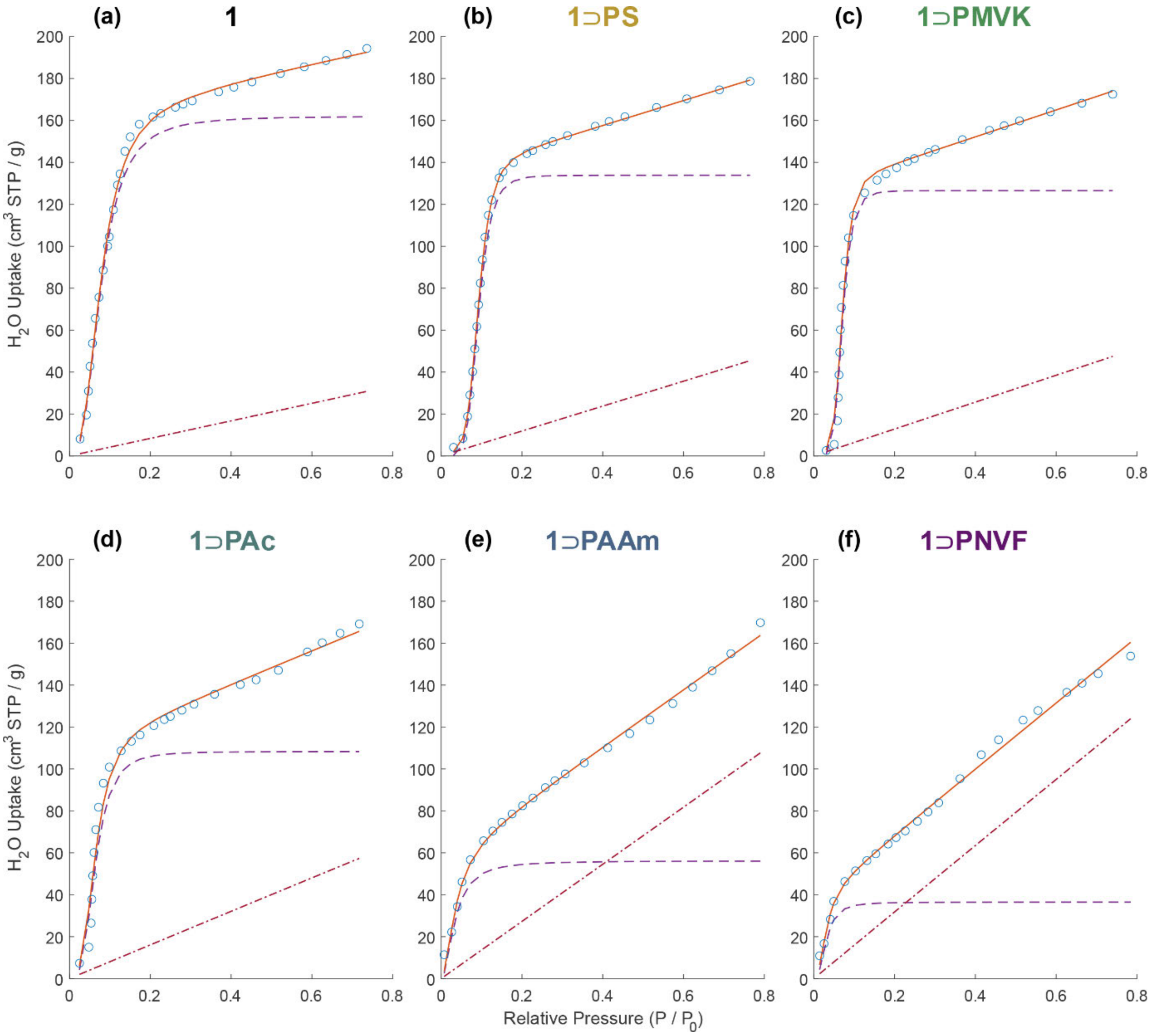

Supplementary Fig. 6. Cooperative adsorption fitting using the method of Dalby *et al.*[2] on H_2O sorption isotherms of (a) **1** and its composites with (b) **PS**, (c) **PMVK**, (d) **PAc**, (e) **PAAm**, (f) **PNVF**. Experimental data (blue circles), fitted cooperative component (purple line), fitted simple surface component (red line), and total fitted isotherm (orange line) are plotted for each composite. The 'step height' (asymptote to which the cooperative component converges) is interpreted as sorption capacity within the micropores, whereas the surface component is attributed to sorption outside the crystallites.

The two-term cooperative sorption isotherm used for the fitting analysis is described as follows.[2]

$$\langle n_2 \rangle = N^{(I)} \frac{A_1^{(I)} a_2}{1 + A_1^{(I)} a_2} + N^{(II)} \frac{A_m^{(II)} a_2^m}{1 + A_m^{(II)} a_2^m}$$

$\langle n_2 \rangle$ is the number of sorbates (uptake), a_2 is taken as P/P_0 from the isotherm, and the terms N, A, and m are fitted parameters. The 'step height', $h = N^{(II)}m$, is the amount of sorption associated with saturation of the cooperative site, which is taken to mean the triangular pore in **1**.

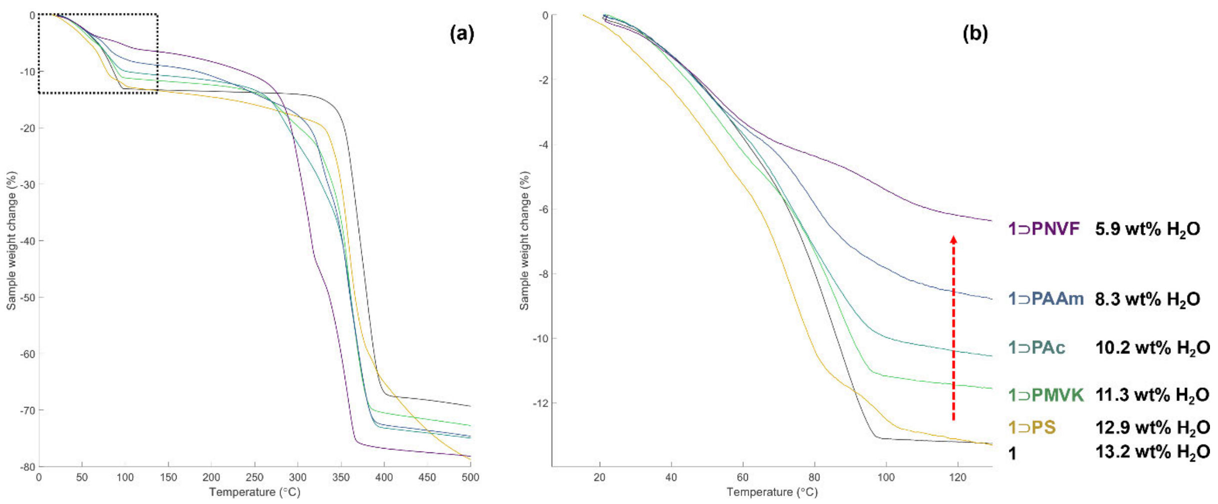

Supplementary Fig. 7. Thermogravimetry (TG) curves of **1** and its composites with **PS**, **PMVK**, **PAc**, **PAAm**, and **PNVF**. (a) Full curves and (b) their expanded view of the first 100 °C of heating. The loss in mass at the 100 °C step is attributed to H_2O desorption, and its magnitude displays the same trend as seen in volumetric H_2O sorption experiments— a monotopic decrease as guest polarity increases. As the dehydration of **1** is the driving force between its phase transition from gate-open to gate-closed, it was inferred from this data that VT-PXRD may be used to observe such transitions and any changes guest polymers cause in them.

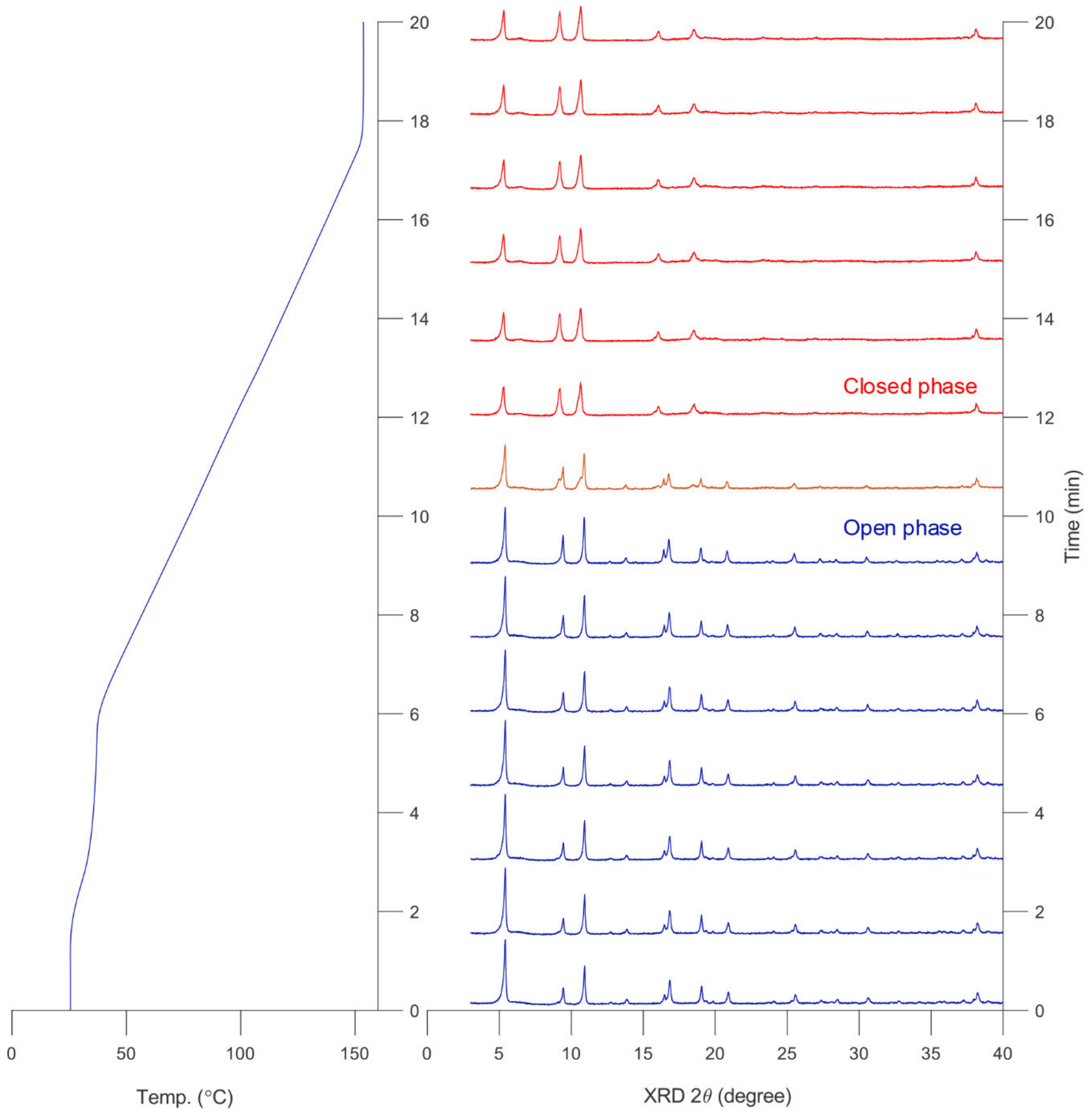

Supplementary Fig. 8. VT-PXRD patterns of **1** upon heating from room temperature to 150 °C. A transition from open (blue)-to-mixed (orange)-to-closed phase (red) is observed upon crossing ~100 °C, which is attributed to H_2O desorption based on the TG curves (Supplementary Fig. 7), structural models, and a prior study of transitions in an isostructural MOF.[1]

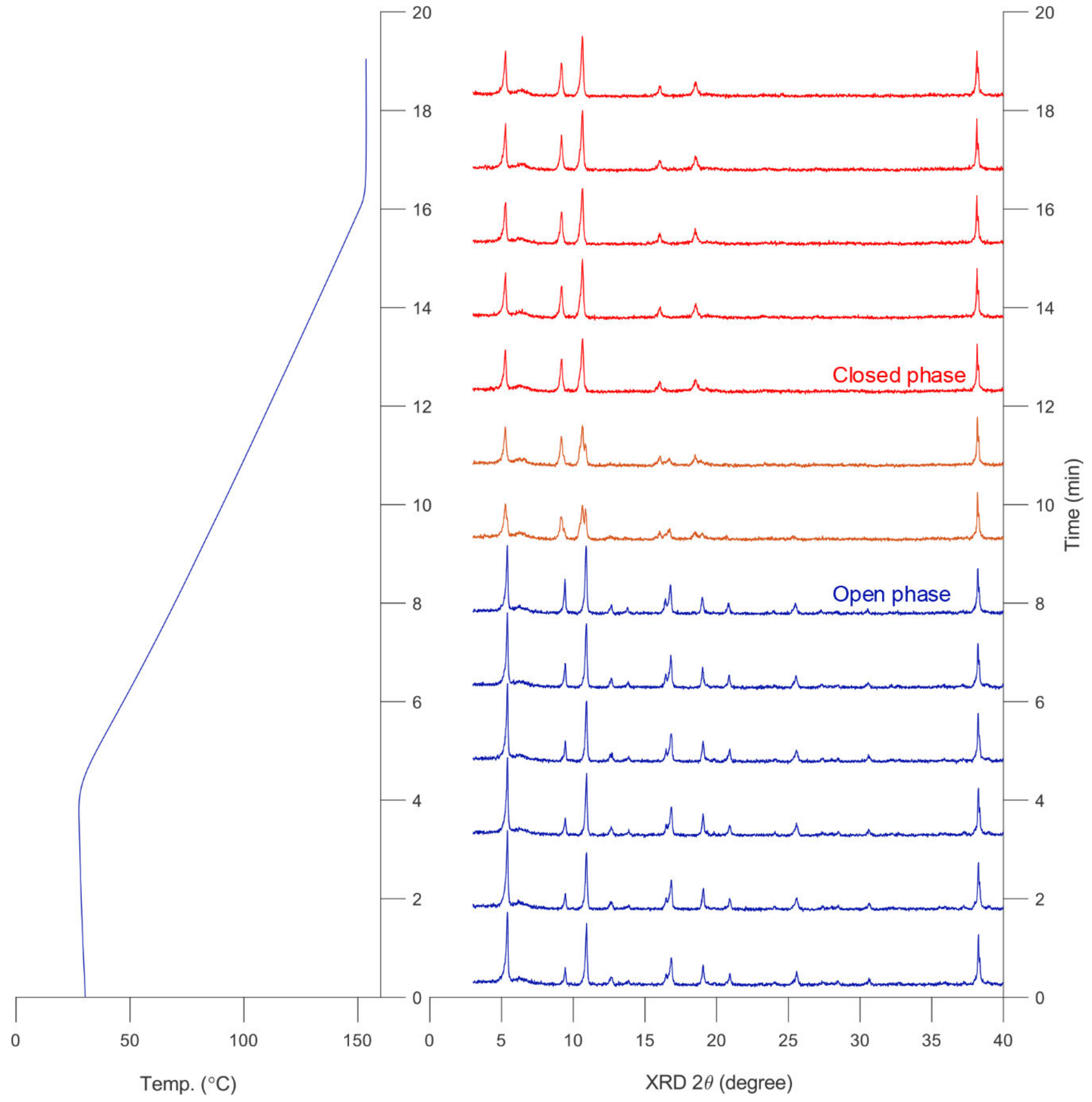

Supplementary Fig. 9. VT-PXRD patterns of **1⊃PS** upon heating from room temperature to 150 °C. Colors highlight open (blue), mixed (orange), and closed (red) phases.

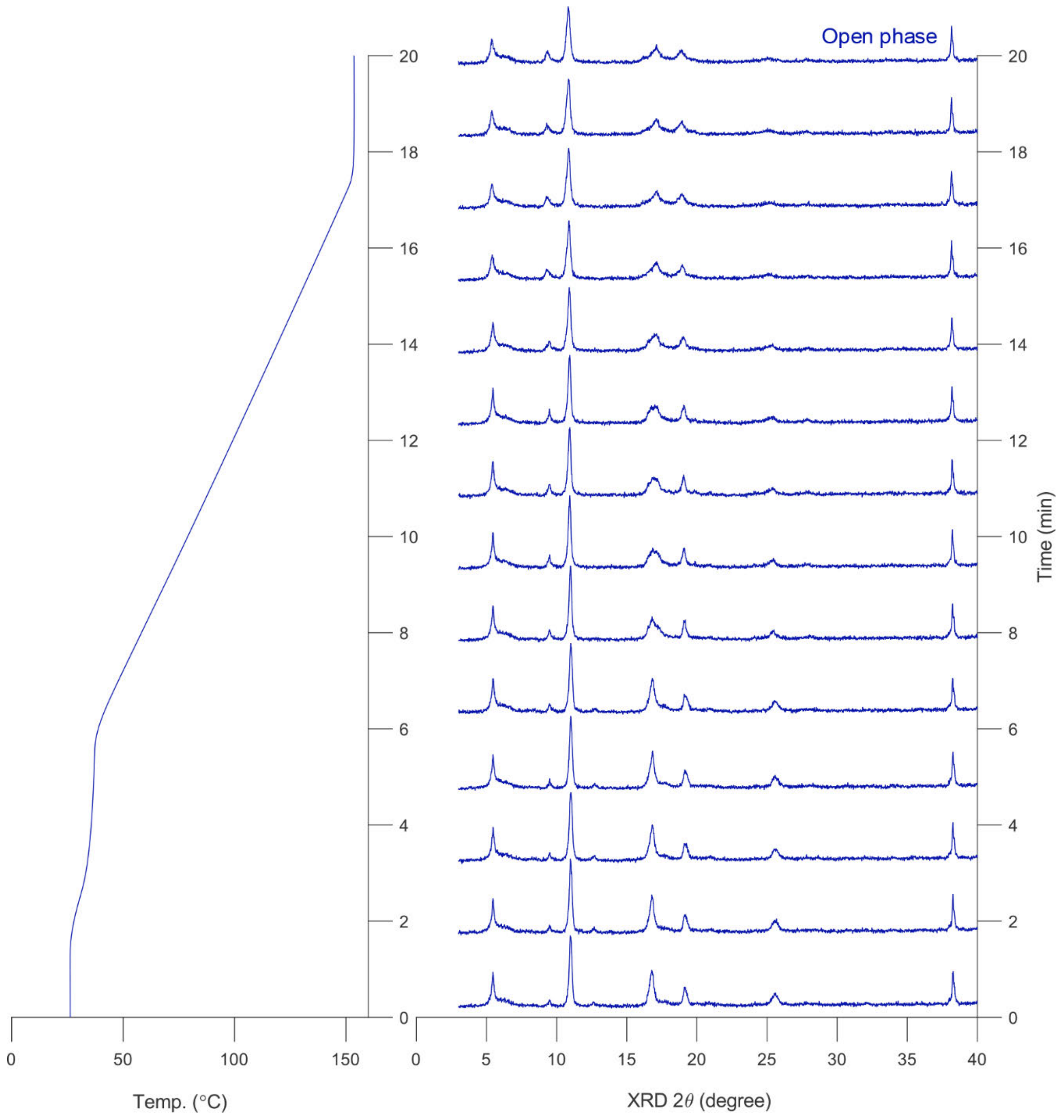

Supplementary Fig. 10. VT-PXRD patterns of **1⊃PNVF** upon heating from room temperature to 150 °C. Interestingly, the phase transition seen for **1⊃PS** has been replaced by a simpler peak broadening effect which retains the open-phase peak positions throughout. This suggests **PNVF** chain has coordinated to Cu(II) open metal sites on the paddle wheel clusters instead of H_2O, 'locking' the phase of the composite as the polymer does not desorb upon heating.

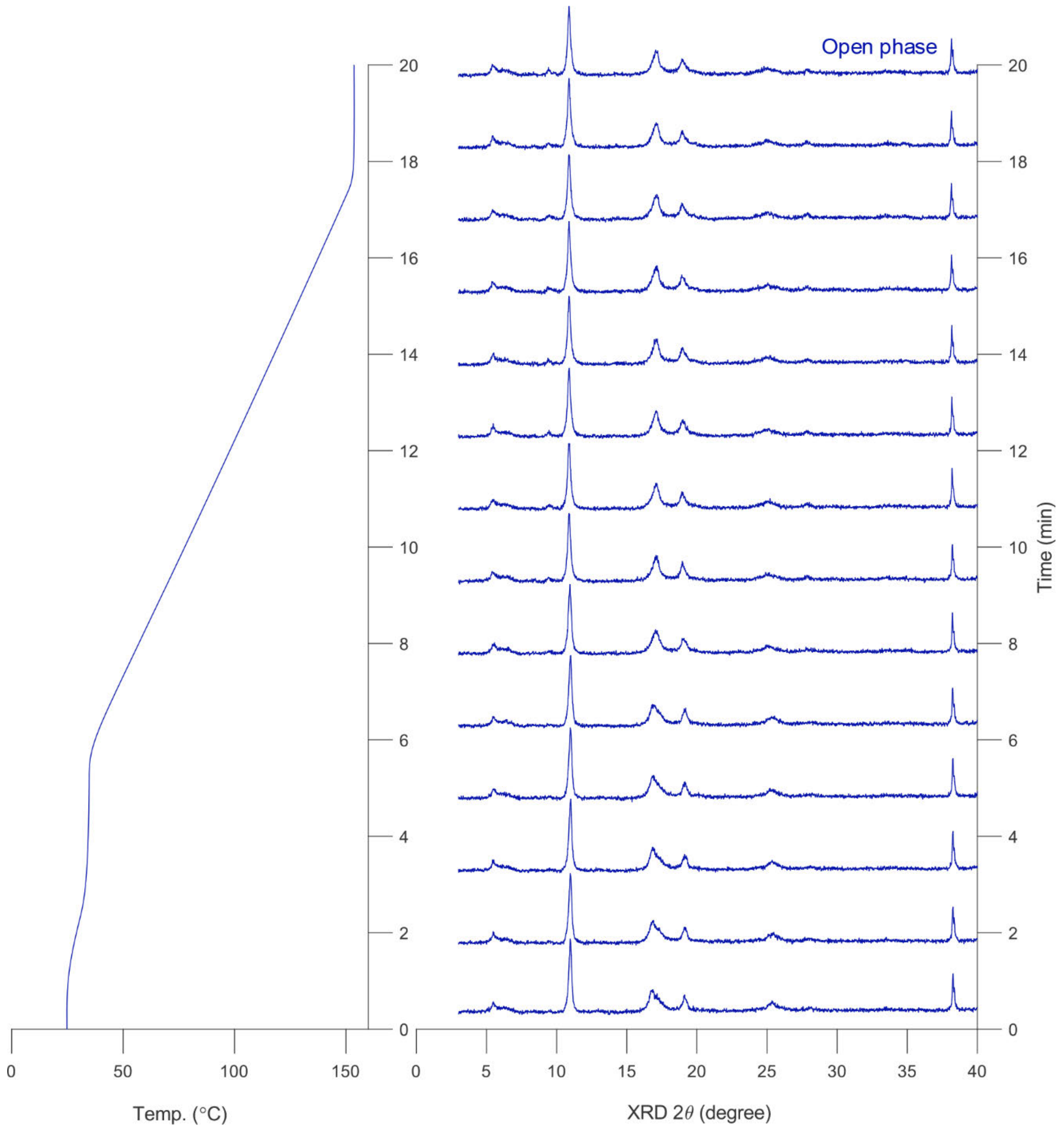

Supplementary Fig. 11. VT-PXRD patterns of simultaneous-insertion **1⊃PS/PNVF** upon heating from room temperature to 150 °C. This composite also displays the phase-locking property seen in **1⊃PNVF** (Supplementary Fig. 10).

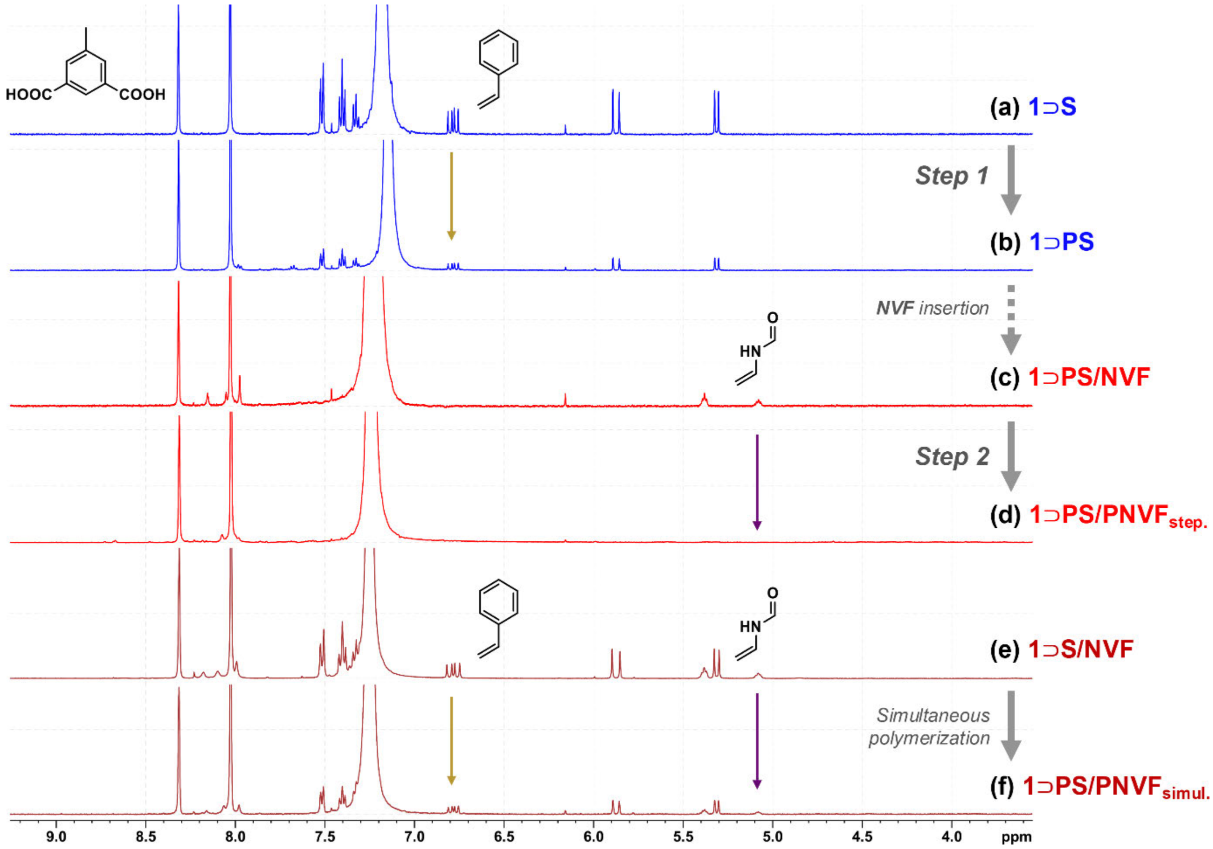

Supplementary Fig. 12. ^{1}H NMR spectra of DMSO-d_6/DCl (9/1, v/v)-digested composites (a) **1⊃S**, (b) **1⊃PS**, (c) **1⊃PS/NVF** and (d) **1⊃PS/PNVF$_{step.}$** synthesized through the stepwise polymerization route, as well as (e) **1⊃S/NVF** and (f) **1⊃PS/PNVF$_{simul.}$** synthesized through the simultaneous polymerization route.

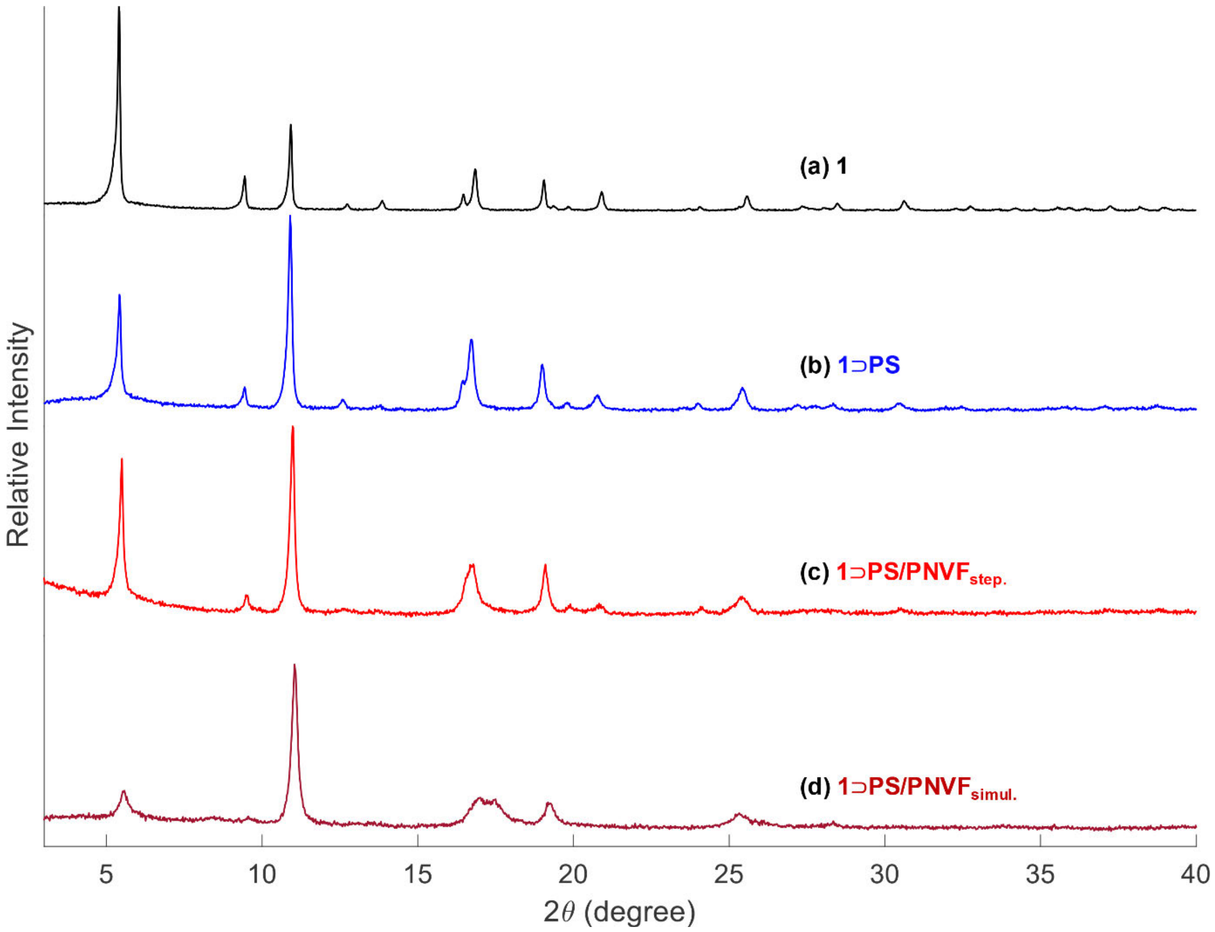

Supplementary Fig. 13. PXRD patterns of (a) **1** compared to (b) **1⊃PS** and (c) **1⊃PS/PNVF**$_{\mathbf{step.}}$ synthesized through the stepwise polymerization route, as well as (d) **1⊃PS/PNVF**$_{\mathbf{simul.}}$ synthesized through the simultaneous polymerization route. As there is no significant difference in polymer loading between the two **1⊃PS/PNVF** samples, peak intensity differences (particularly in low-angle peaks) are attributed to a slight excess of unreacted monomer present in the stepwise but absent in the simultaneous route sample.

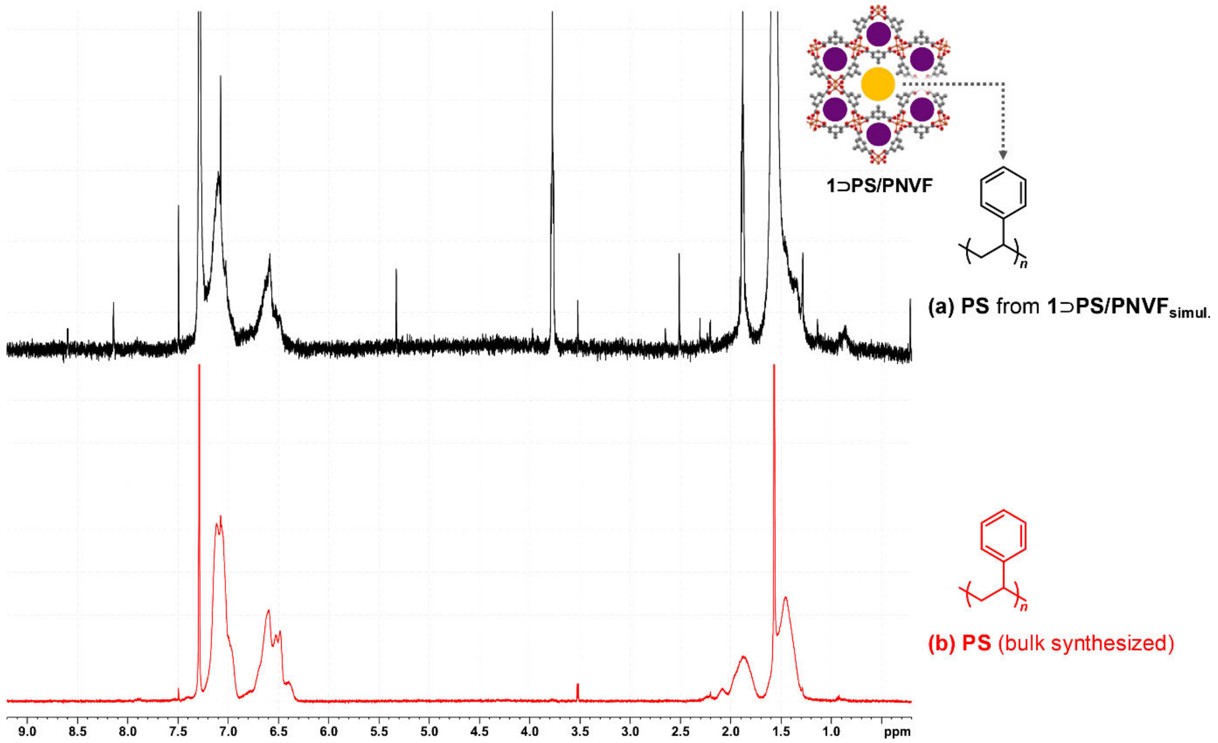

Supplementary Fig. 14. ^{1}H NMR ($CDCl_3$) spectrum of (a) **PS** isolated from **1⊃PS/PNVF$_{simul.}$** synthesized through the simultaneous polymerization route, compared to (b) bulk-synthesized polystyrene homopolymer. Matching spectra and a lack of amide peaks from **PNVF** support that styrene underwent homopolymerization within **1**. **PS** was extracted from **1** by dissolving the composite in HCl solution, then precipitating the polymer from the resulting solution with methanol.

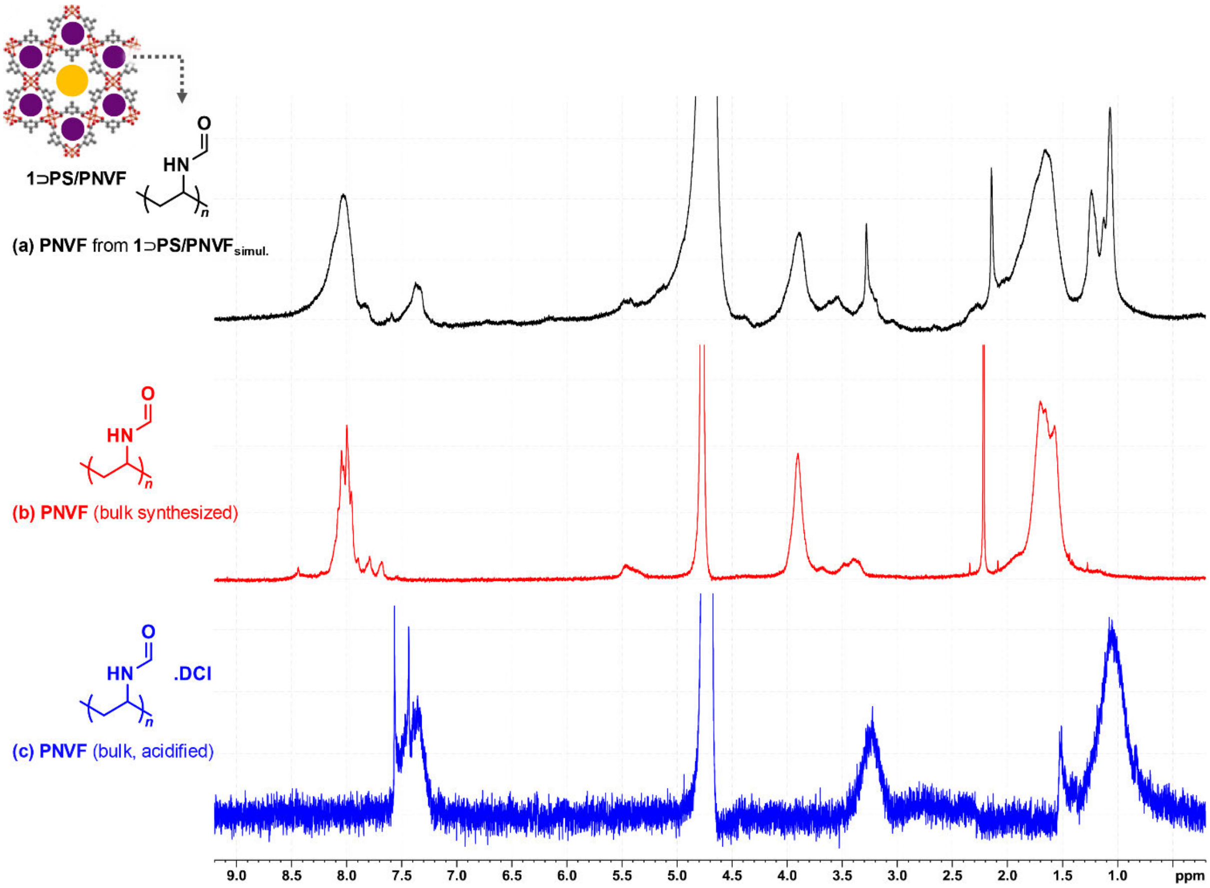

Supplementary Fig. 15. ^{1}H NMR (D_2O) spectrum of (a) **PNVF** isolated from **1⊃PS/PNVF$_{\textbf{simul.}}$** synthesized through the simultaneous polymerization route, compared to (b) bulk-synthesized PNVF homopolymer and (c) the same bulk PNVF acidified in DCl. The polymer extracted from **1** displays peaks from both (b) and (c), indicating a partially acidified form of **PNVF**, likely the result of the HCl used to dissolve **1**. Nevertheless, all polymeric peaks in (a) are accounted for in (b, c), and a lack of phenyl peaks supports that **NVF** homopolymerized within **1**. **PNVF** was extracted from **1** by dissolving the composite in HCl solution, then precipitating the polymer from the resulting solution with acetone.

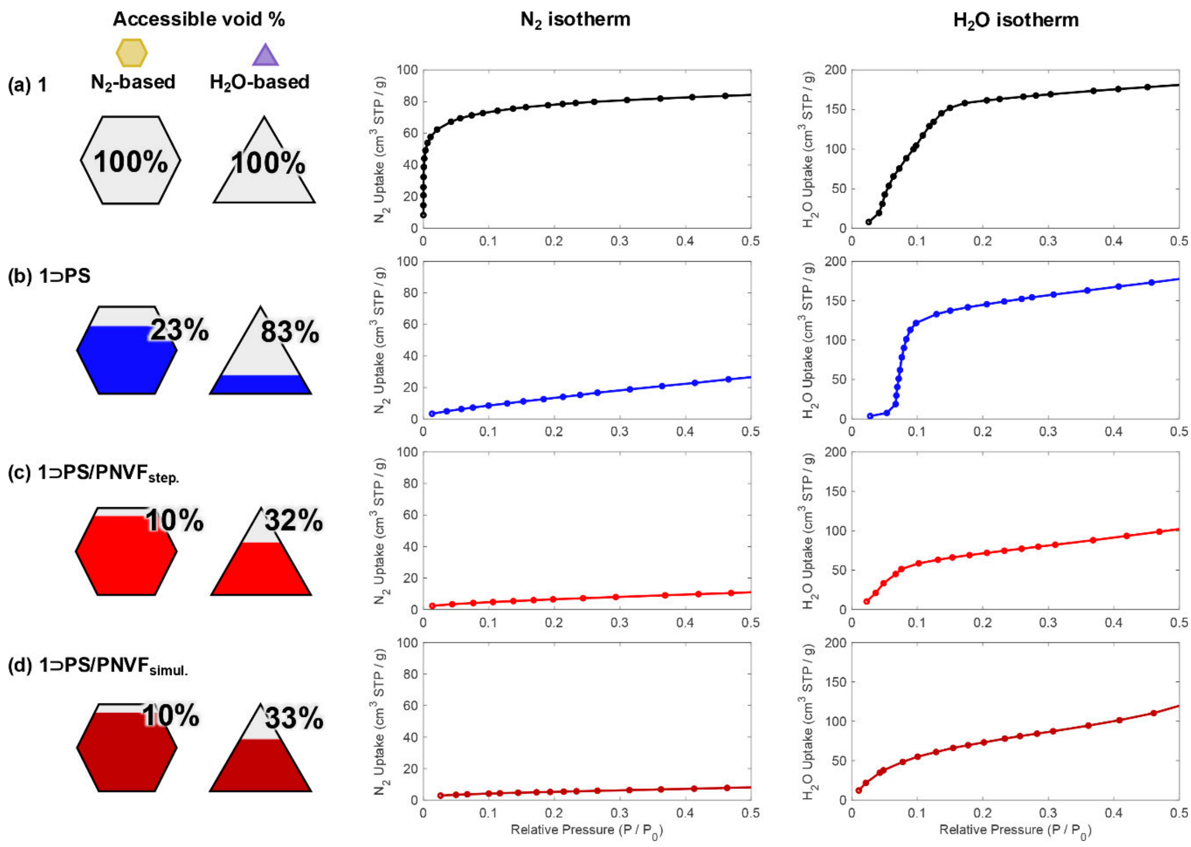

Supplementary Fig. 16. Accessible void percentages (left) and N_2 (center) and H_2O (right) sorption isotherms of (a) **1** compared to (b) **1⊃PS** and (c) **1⊃PS/PNVF$_{\mathbf{step.}}$** synthesized through the stepwise polymerization route, as well as (d) **1⊃PS/PNVF$_{\mathbf{simul.}}$** synthesized through the simultaneous polymerization route. Loss of micropore capacity seen in the low-p/p_0 regions of both indicates filling of both pores by polymer. Like with PXRD, while this suggests the filling of both pores with polymers, the distribution (i.e. location) of each polymer species within the crystal cannot be inferred. Hence, the stepwise polymerization experiment was conducted.

Regarding the H_2O adsorption profiles, the gate-opening behavior, signified by a sigmoidal isotherm profile, is observed in (a) **1** and (b) **1⊃PS**, but not for (c) **1⊃PS/PNVF$_{\mathbf{step.}}$** and (d) **1⊃PS/PNVF$_{\mathbf{simul.}}$** The closed-to-open transformation of **1** is induced by the coordination of H_2O molecules to the Cu open metal sites located within the triangular pores of the MOF.[1] In **1⊃PS**, the triangular pores remain accessible, as PS chains occupy only the hexagonal pores. Thus, this composite shows gate-opening behavior as seen in empty **1**. In contrast, both **1⊃PS/PNVF$_{\mathbf{step.}}$** and **1⊃PS/PNVF$_{\mathbf{simul.}}$** exhibit significantly reduced H_2O uptake and do not display typical gate-type isotherms, due to the occupation of the triangular pores by PNVF chains. The coordination of PNVF to the Cu open-metal sites stabilizes the open-phase structure, preventing coordination of H_2O and disabling dynamic gate-opening behavior by keeping **1** open permanently.

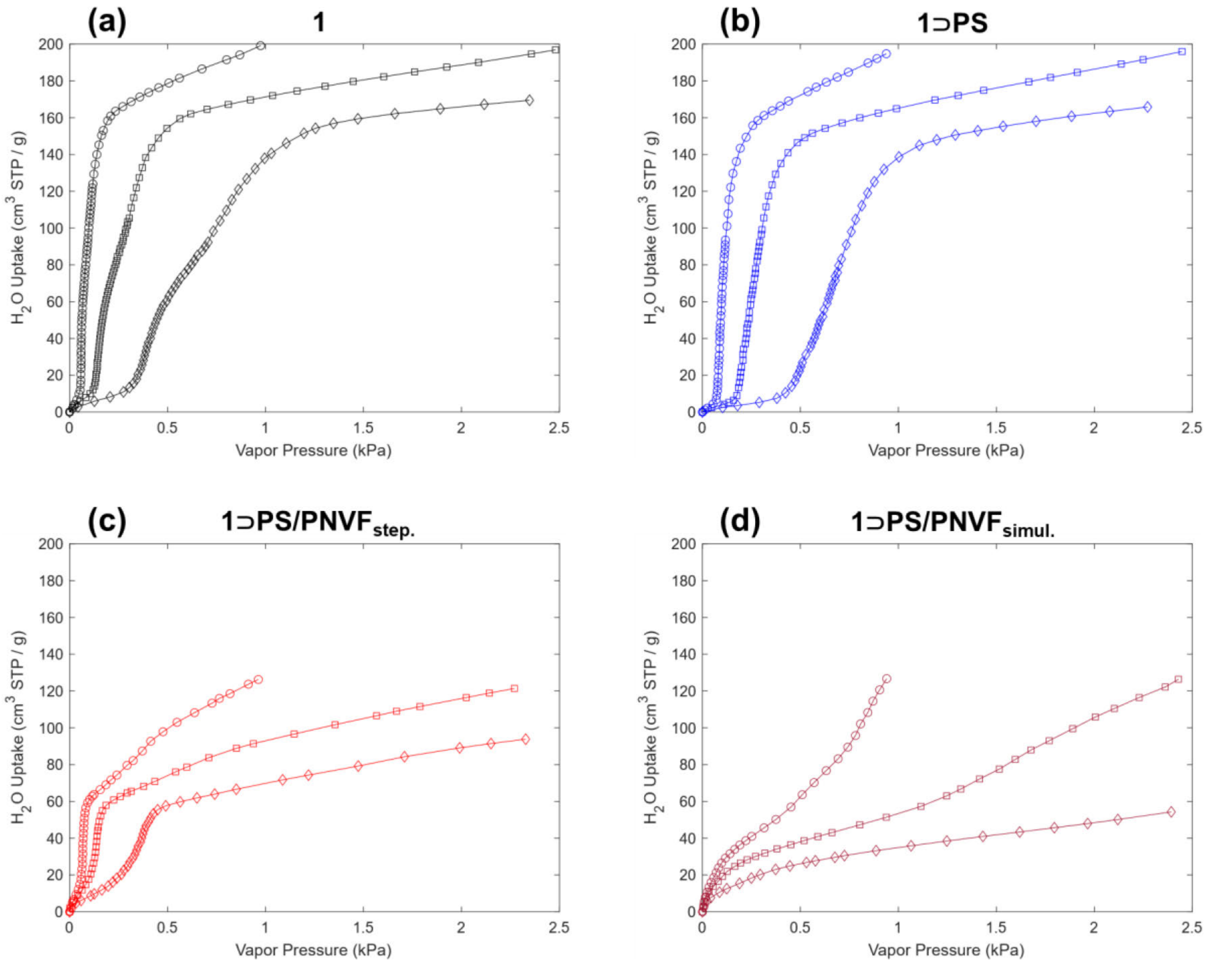

Supplementary Fig. 17. H_2O adsorption isotherms measured at 10 °C (circles), 25 °C (squares), 40 °C (diamonds) and their linear interpolants (lines) for (a) **1**, (b) **1⊃PS**, (c) **1⊃PS/PNVF$_{step}$**, (d) **1⊃PS/PNVF$_{simul}$**. These isotherms were used for the determination of the isosteric enthalpy of adsorption of water.

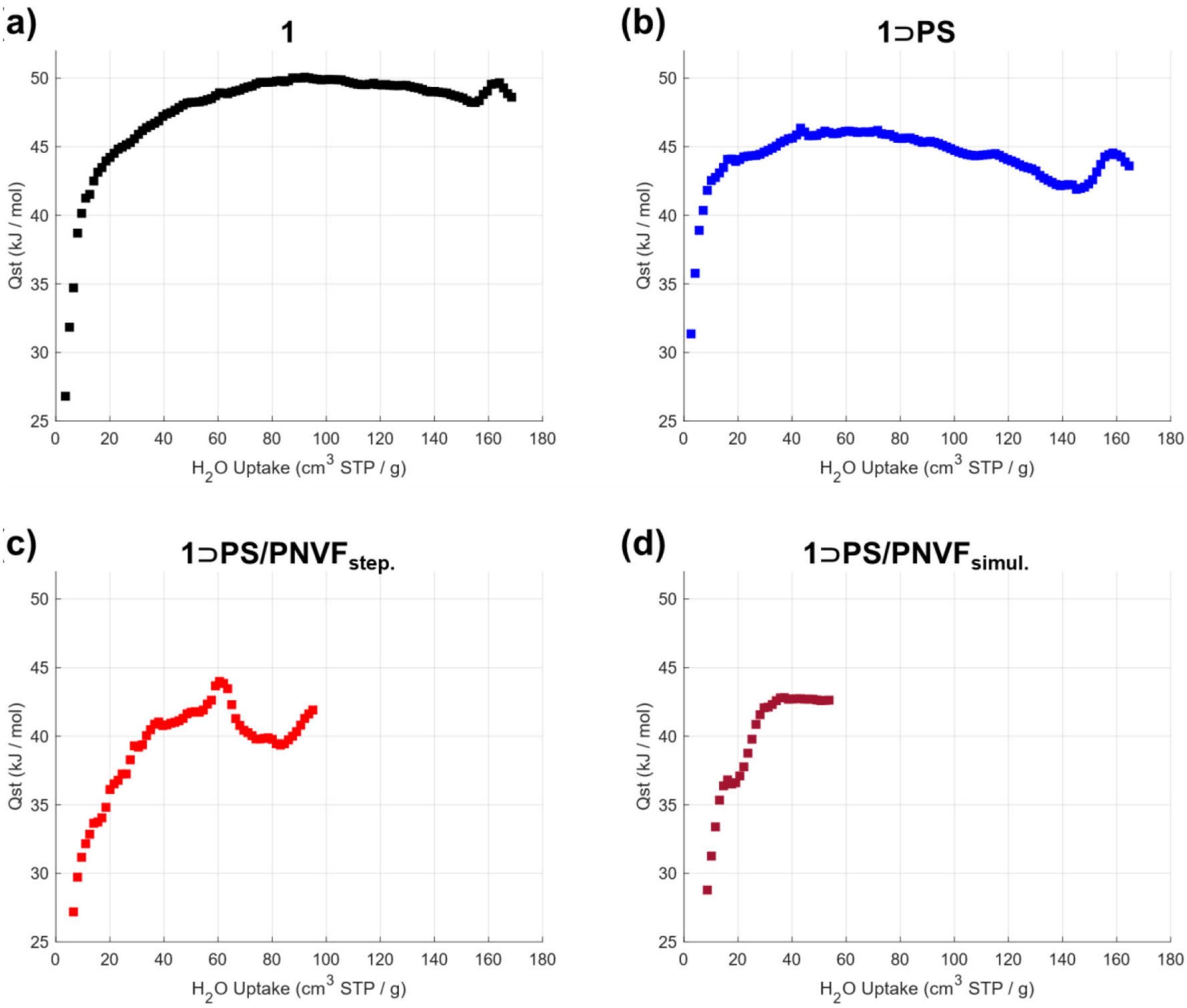

Supplementary Fig. 18. Isosteric heat of adsorption (Q_{st}) of H_2O derived by fitting of the Clausius-Clapeyron equation for sorption isotherms of (a) **1**, (b) **1⊃PS**, (c) **1⊃PS/PNVF$_{step}$**, (d) **1⊃PS/PNVF$_{simul}$**. Fits were carried out only for uptake values where data could be interpolated for all three temperatures (10, 25, and 40 °C). A sharp peak originating from increased hydrogen bond formation is observed at the uptake where triangular pores become saturated, corroborating that the change in available volume in these pores is negligible upon introduction of PS (164 → 159 cm^3 (STP)/g), but large (164 → 60 cm^3 (STP)/g) for PNVF. This supports the site selectivity of the two species.

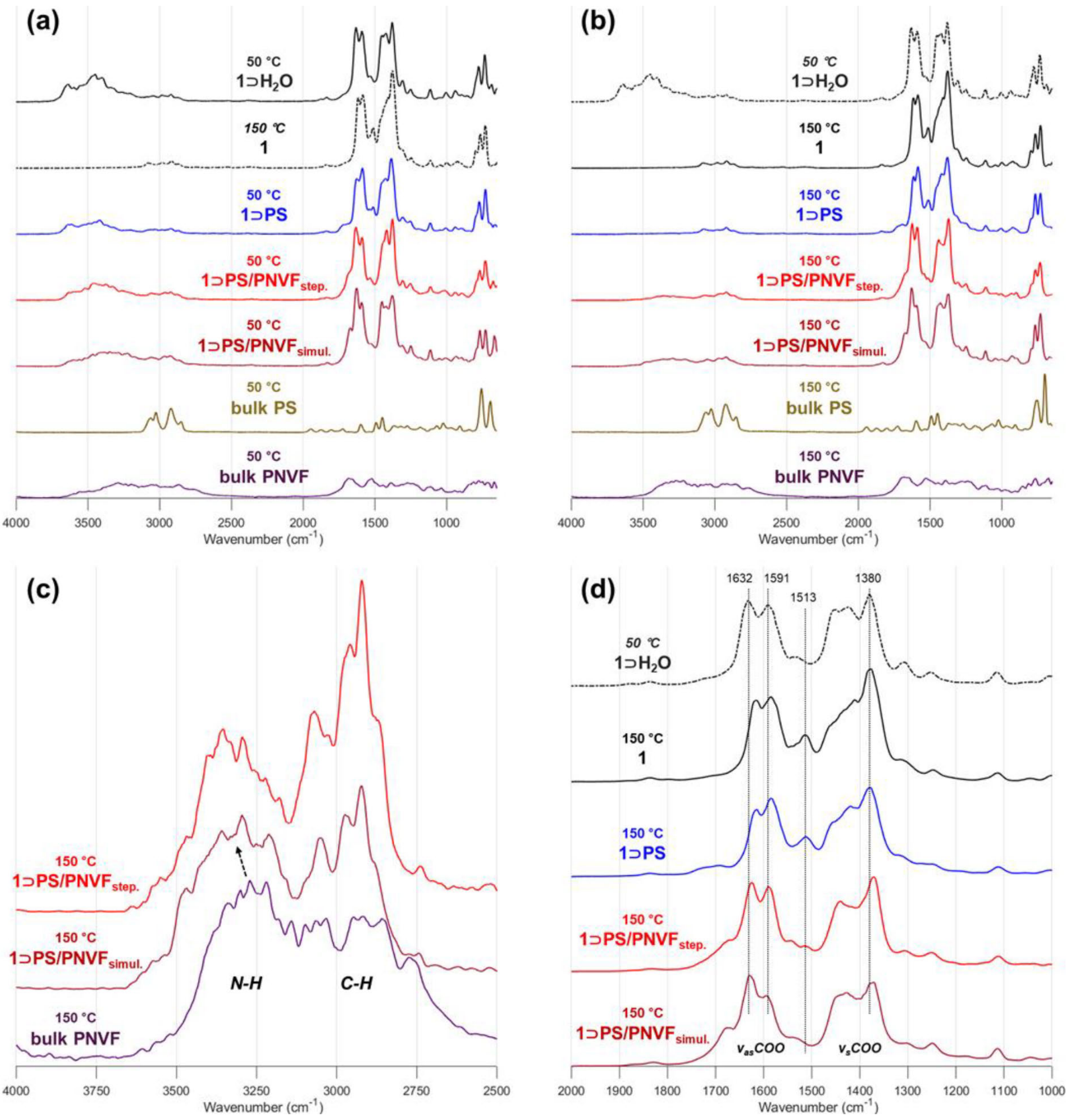

Supplementary Fig. 19. Variable-temperature diffuse reflectance FT-IR (DRIFTS) spectra of composites **1⊃PS**, **1⊃PS/PNVF$_{step}$**, **1⊃PS/PNVF$_{simul}$**, PS, and PNVF. (a) Initial spectra at 50 °C. (b) Final spectra at 150 °C. Both the gate-open (**1**⊃H_2O at 50 °C) and gate-closed (**1** at 150 °C) forms of the MOF are provided for comparison. Magnified views are provided for comparison of (c) the N-H stretching band in bulk PNVF vs. **1⊃PS/PNVF** composites, and (d) COO^{-} stretches of various composites of **1**.

Note that while H_2O is present in all composites at 50 °C, these are fully dehydrated by 150 °C as evidenced by the loss of O-H stretching bands. Compounds which transition to gate-closed phase additionally experience a red-shift of ν_{as}COO^{-} and display an additional characteristic band at 1513 cm^{-1} upon dehydration. Composites containing PNVF exhibit similar spectra to the H_2O-coordinated form of **1** even when dehydrated, indicating a similar axially-coordinated environment around Cu and, coupled with a shift in the N-H band, show that PNVF's amide moiety coordinates to Cu in place of the typical H_2O. This explains both the self-sorting behavior of NVF and the "gate-locking" behavior in composites of **1** containing PNVF (Supplementary Figs. 8-11).

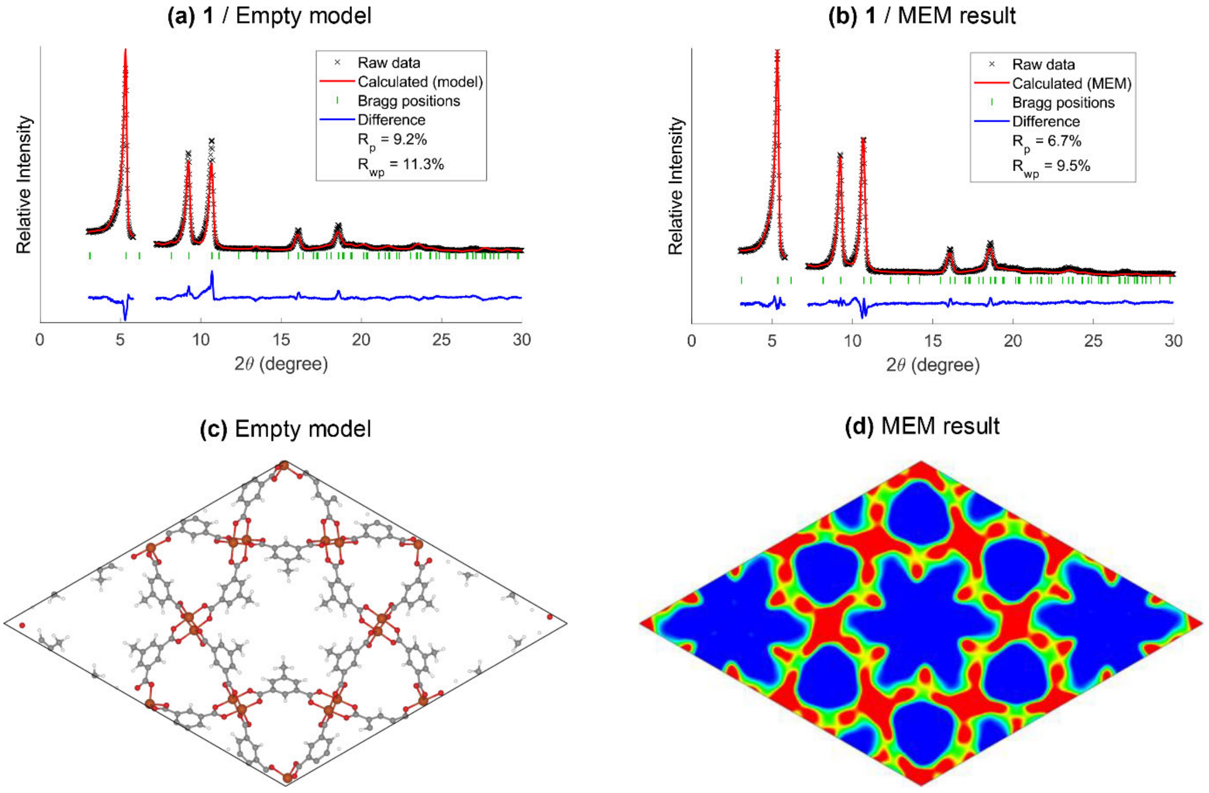

Supplementary Fig. 20. Rietveld plots comparing experimental capillary PXRD data of vacuum-sealed (guest-free) **1** to patterns simulated from (a) the DFT-optimized atomistic closed-phase model of **1**, (b) the structure factors resulting from MEM analysis. The corresponding 3D structures from which calculated patterns were derived are displayed as projections along the crystallographic *c*-axis for reference: (c) the closed-phase DFT model, (d) the MEM electron density map. Little difference in structure or *R*-factors is seen between the two as the empty atomistic model is already a sufficient representation of the sample in this case, and the MEM result corroborates this. $2\theta \sim 6\text{-}7°$ was excluded from fitting procedures to avoid fitting artifacts resulting from the presence of an anomalous peak near the Bragg position in this region.

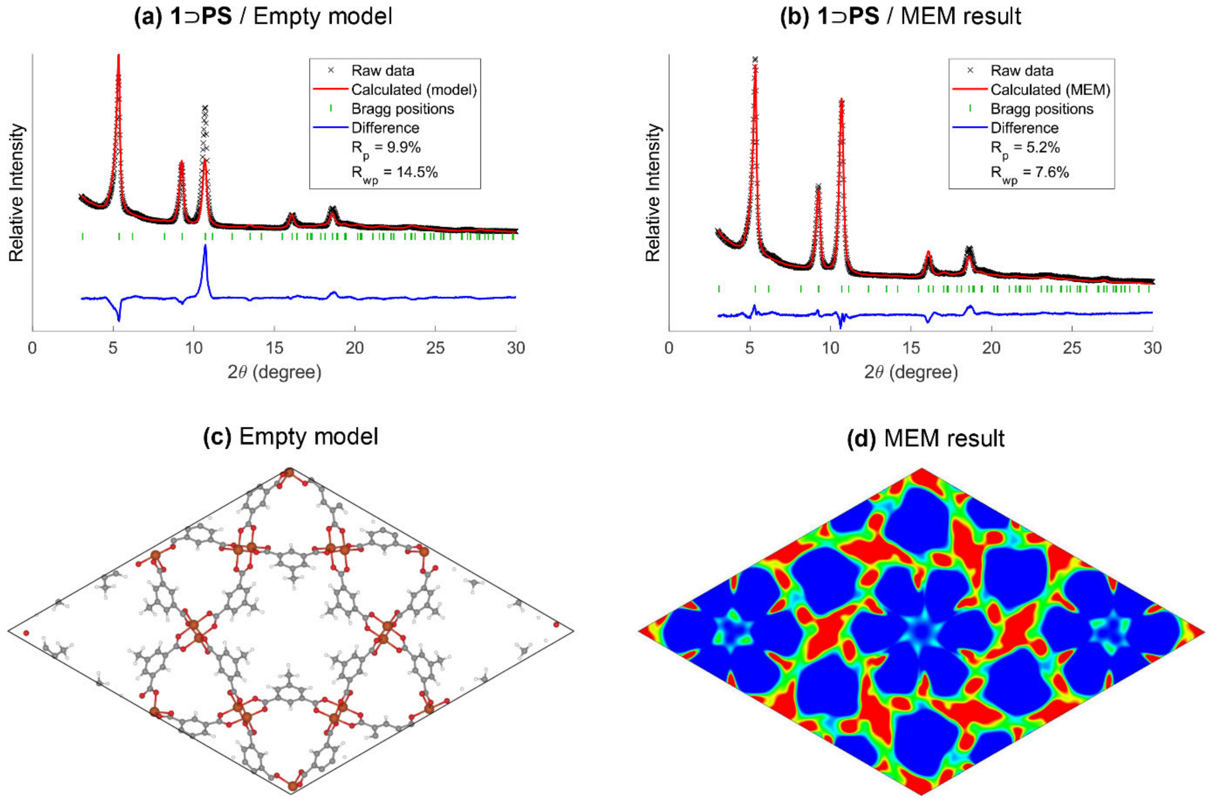

Supplementary Fig. 21. Rietveld plots comparing experimental capillary PXRD data of vacuum-sealed **1⊃PS** to patterns simulated from (a) the DFT-optimized atomistic closed-phase model of **1**, (b) the structure factors resulting from MEM analysis. The corresponding 3D structures from which calculated patterns were derived are displayed as projections along the crystallographic *c*-axis for reference: (c) the closed-phase DFT model, (d) the MEM electron density map. Considerable improvement of the PXRD pattern fit upon application of MEM analysis indicates that the increased electron density within the hexagonal pores is structurally significant and better represents the density distribution in the **1⊃PS** sample. The appearance of electron density in only the hexagonal pores is attributed to the formation of **PS** exclusively in these pores, and is supported by the sorption results seen in Supplementary Figure 16.

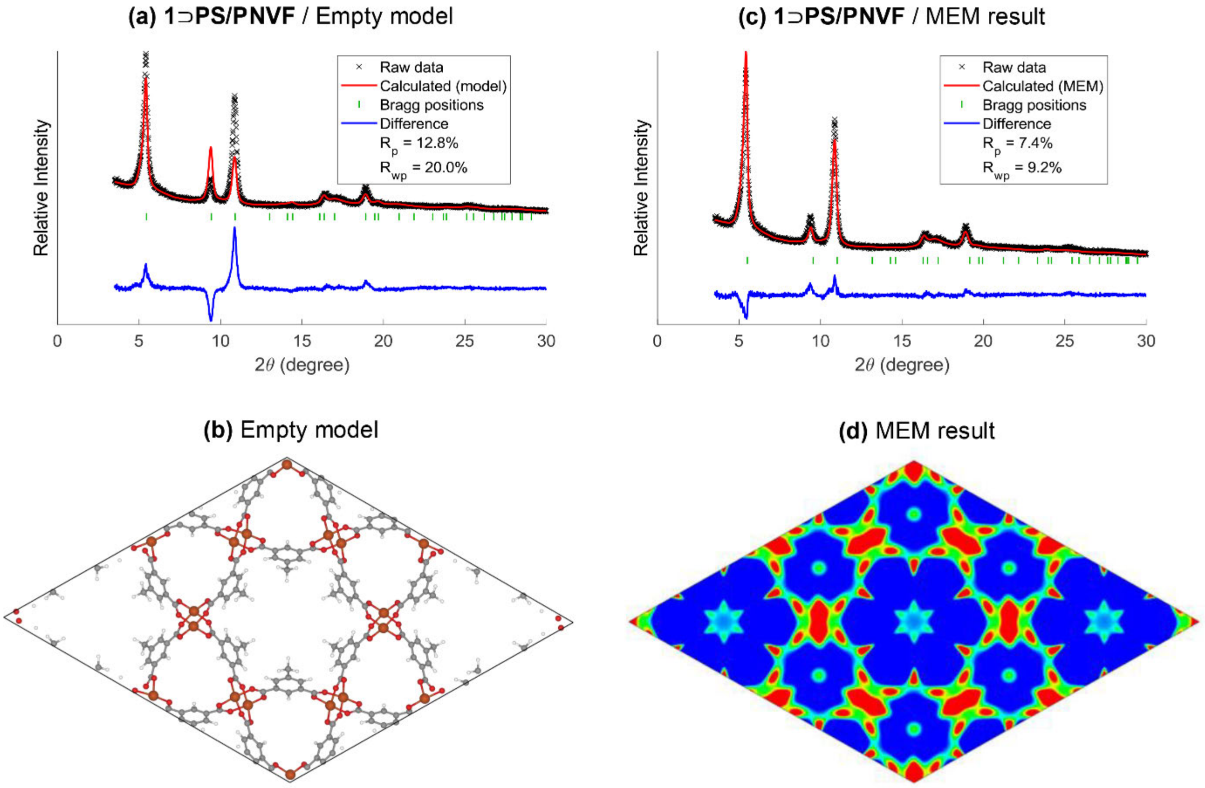

Supplementary Fig. 22. Rietveld plots comparing experimental capillary PXRD data of vacuum-sealed **1⊃PS/PNVF** to patterns simulated from (a) the DFT-optimized atomistic open-phase model of **1**, (b) the structure factors resulting from MEM analysis. The corresponding 3D structures from which calculated patterns were derived are displayed as projections along the crystallographic *c*-axis for reference: (c) the open-phase DFT model, (d) the MEM electron density map. Considerable improvement of the PXRD pattern fit upon application of MEM analysis indicates that the increased electron density within the pores is structurally significant and better represents the density distribution in the **1⊃PS/PNVF** sample. While electron density in the hexagonal pores was already observed and attributed to **PS** in the previous step, additional density is now incorporated in the triangular pores following NVF polymerization and is thus attributed to the presence of **PNVF** in these pores. This is again supported by the sorption results seen in Supplementary Figure 16.

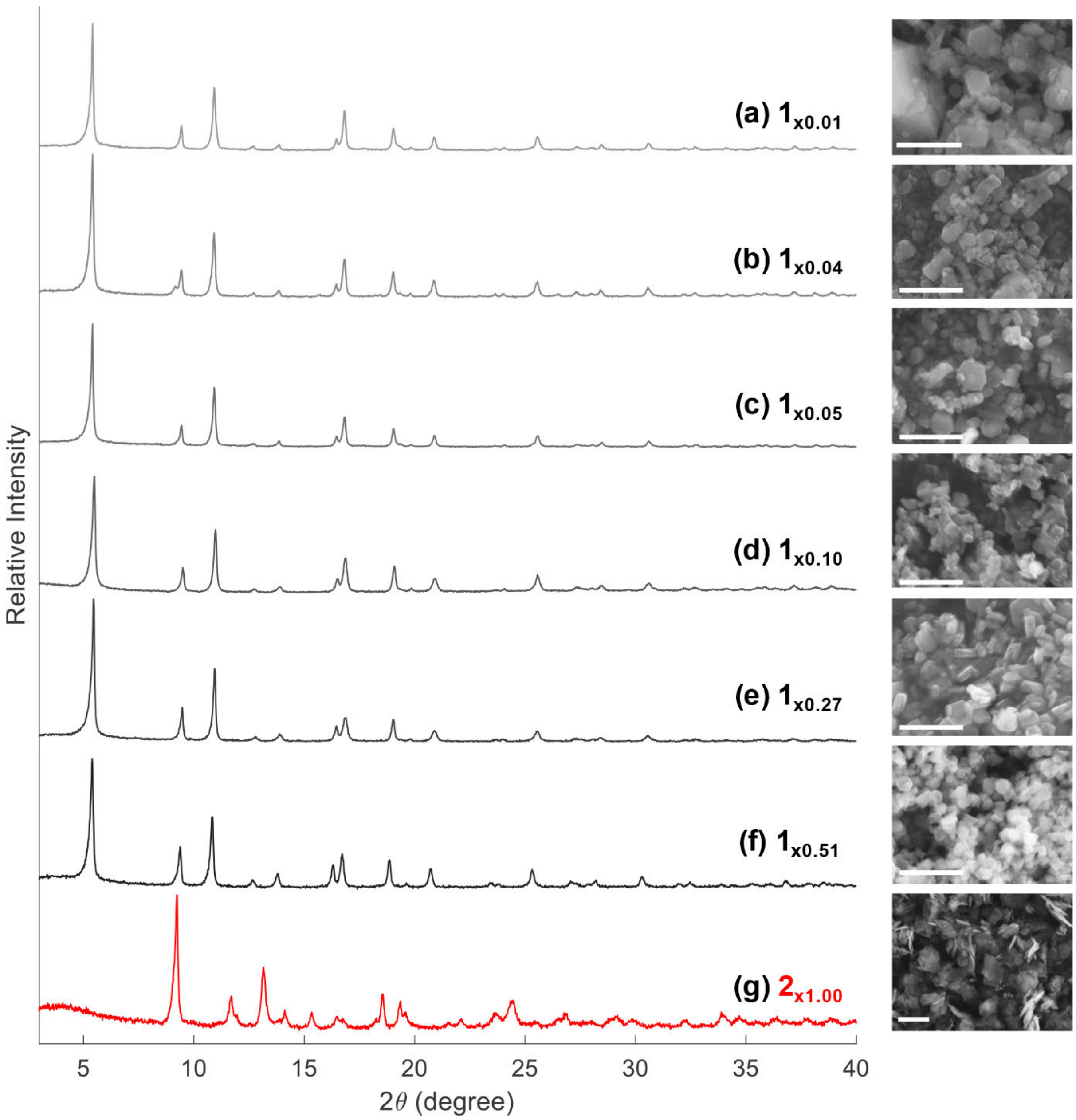

Supplementary Fig. 23. PXRD patterns and SEM images of (a-f) $\mathbf{1}_{xc}$ and (g) $\mathbf{2}_{x1.00}$ (a crystalline nonporous product). Scale bars are 1 μm. Similar XRD patterns and hexagonal platelet morphology are observed up to and including c = 0.51, indicating the same basic structure as **1**. However, use of only dvip results in a crystalline nonporous substance, referred to as $\mathbf{2}_{x1.00}$, consisting of a mixture of needle-like and block-like morphologies. This indicates an upper limit on the amount of dvip that may be incorporated into the MOF.

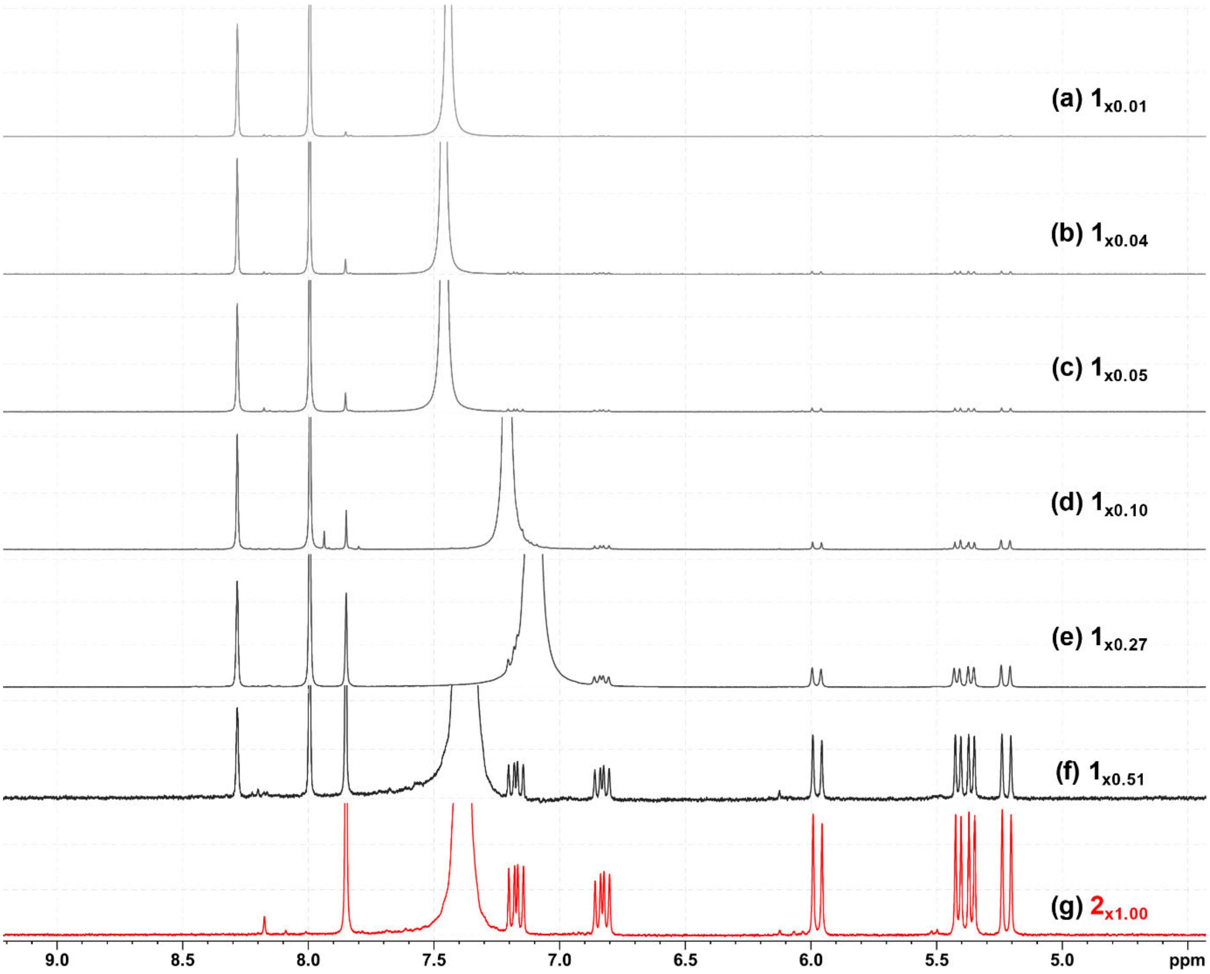

Supplementary Fig. 24. ^{1}H NMR of (a-f) $\mathbf{1}_{\mathrm{x}c}$ (c = 0.01, 0.04, 0.05, 0.10, 0.27, and 0.51) and (g) $\mathbf{2}_{\mathrm{x1.00}}$ digested in DMSO-d_6/DCl (9/1, v/v), showing incorporation of dvip.

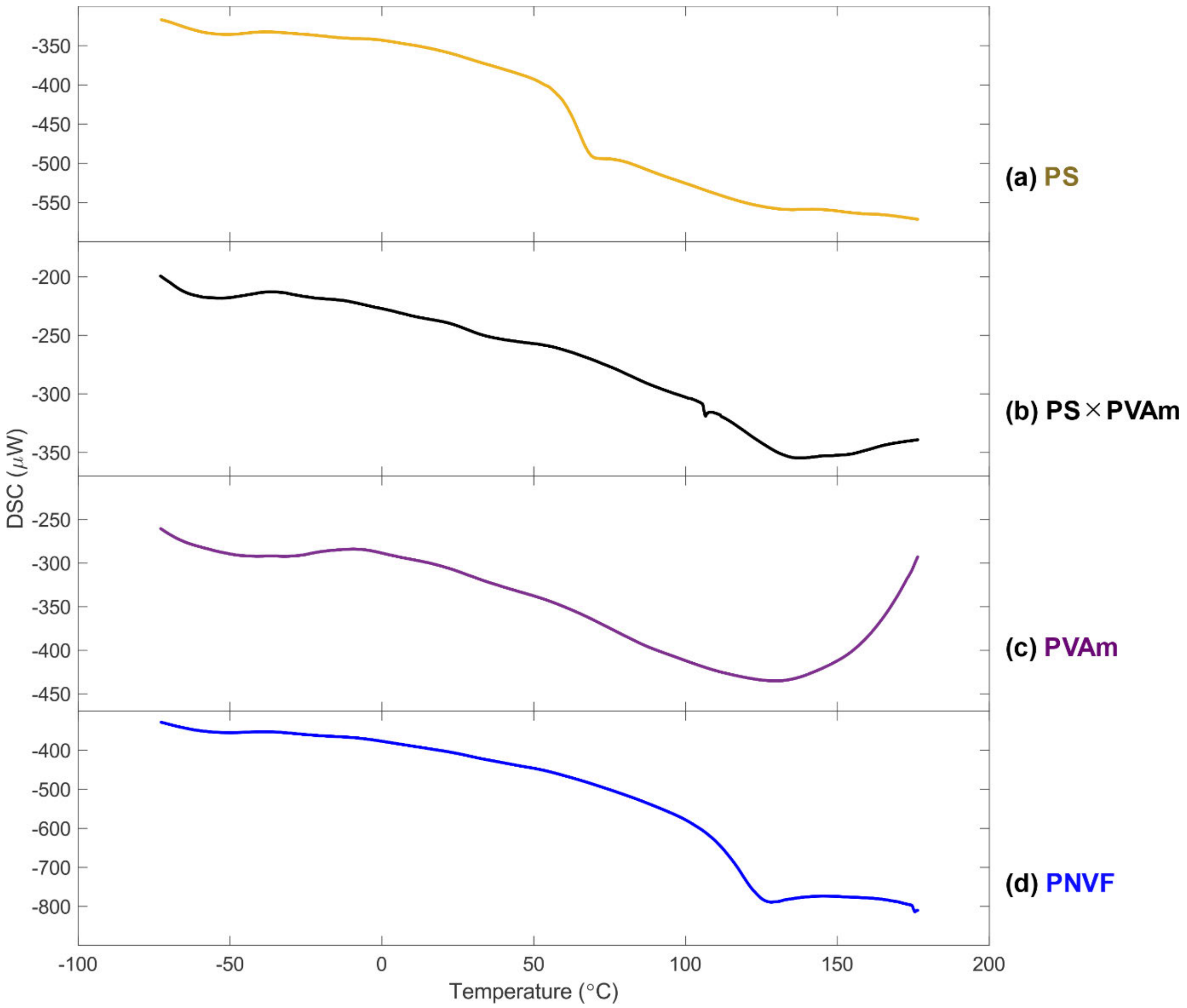

Supplementary Fig. 25. DSC curves of (a) bulk **PS**, (b) **PS×PVAm**, (c) bulk **PVAm** (>90% hydrolysed **PNVF**), (d) bulk **PNVF**. The curves shown are the heating segment of the third of three cycles from –100 to 200 °C. **PS×PVAm** displays no notable glass transition, indicating its component polymers are incapable of undergoing phase separation to form bulk domains, likely due to the presence of crosslinkers between the two throughout their chains.

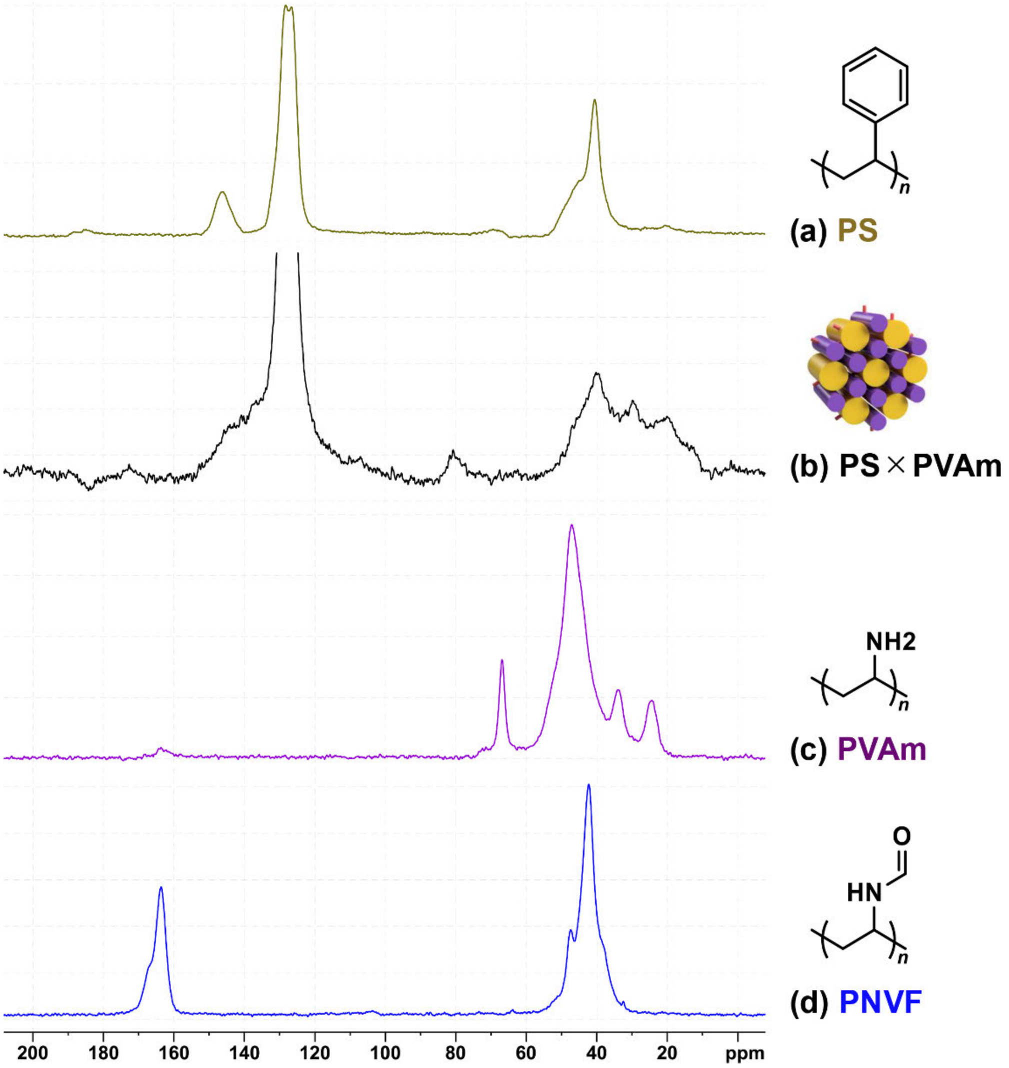

Supplementary Fig. 26. Solid-state ^{13}C-{^{1}H} CP-TOSS NMR of (a) bulk **PS**, (b) **PS×PVAm**, (c) bulk **PVAm** (>90% hydrolyzed **PNVF**), (d) bulk **PNVF**. The peaks of **PS×PVAm** best match a combination of **PS** and **PNVF**, confirming the incorporation of both monomers into the polymer network, as well as the efficient post-synthetic hydrolysis of formamide groups. Note that the most downfield peak of **PS** is shifted upfield to form a shoulder on the peak near 130 ppm, an effect seen elsewhere in crosslinked **PS**.[3] The **PVAm** peaks near 80, 30, and 20 ppm are also shifted up- or downfield relative to their homopolymer equivalents, which may also be due to crosslinking or interaction with the other components.

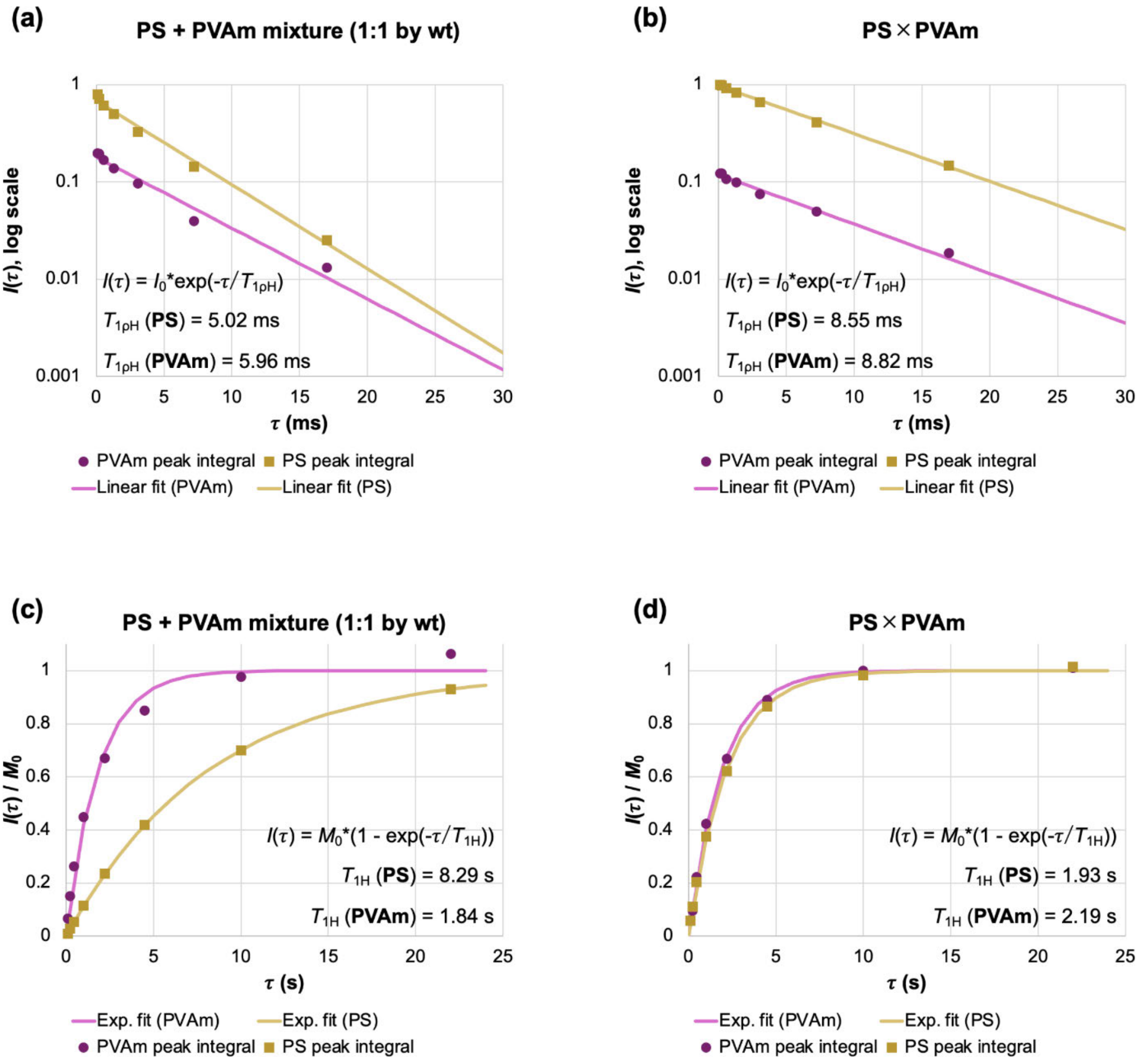

Supplementary Fig. 27. Solid-state NMR ^{1}H relaxation plots of (a, b) $T_{1\rho H}$ and (c, d) T_{1H} experiments for a 1:1 (by weight) mixture of bulk **PS+PVAm**, and for **PS×PVAm** respectively. Proton relaxations of each were measured indirectly by ^{13}C detection via cross-polarization, with T_{1H} measured by saturation recovery. Relaxation curves of characteristic peaks of **PS** in the aromatic region (~130 ppm) and **PVAm** in the upfield region (~20 ppm) were taken to represent each polymer, respectively. Unlike the bulk mixture, **PS×PVAm** displays near-identical $T_{1\rho H}$ and T_{1H} for the two components. This indicates a molecular-level proximity of the two polymers, suggesting the single-chain alternating array structure.[4]

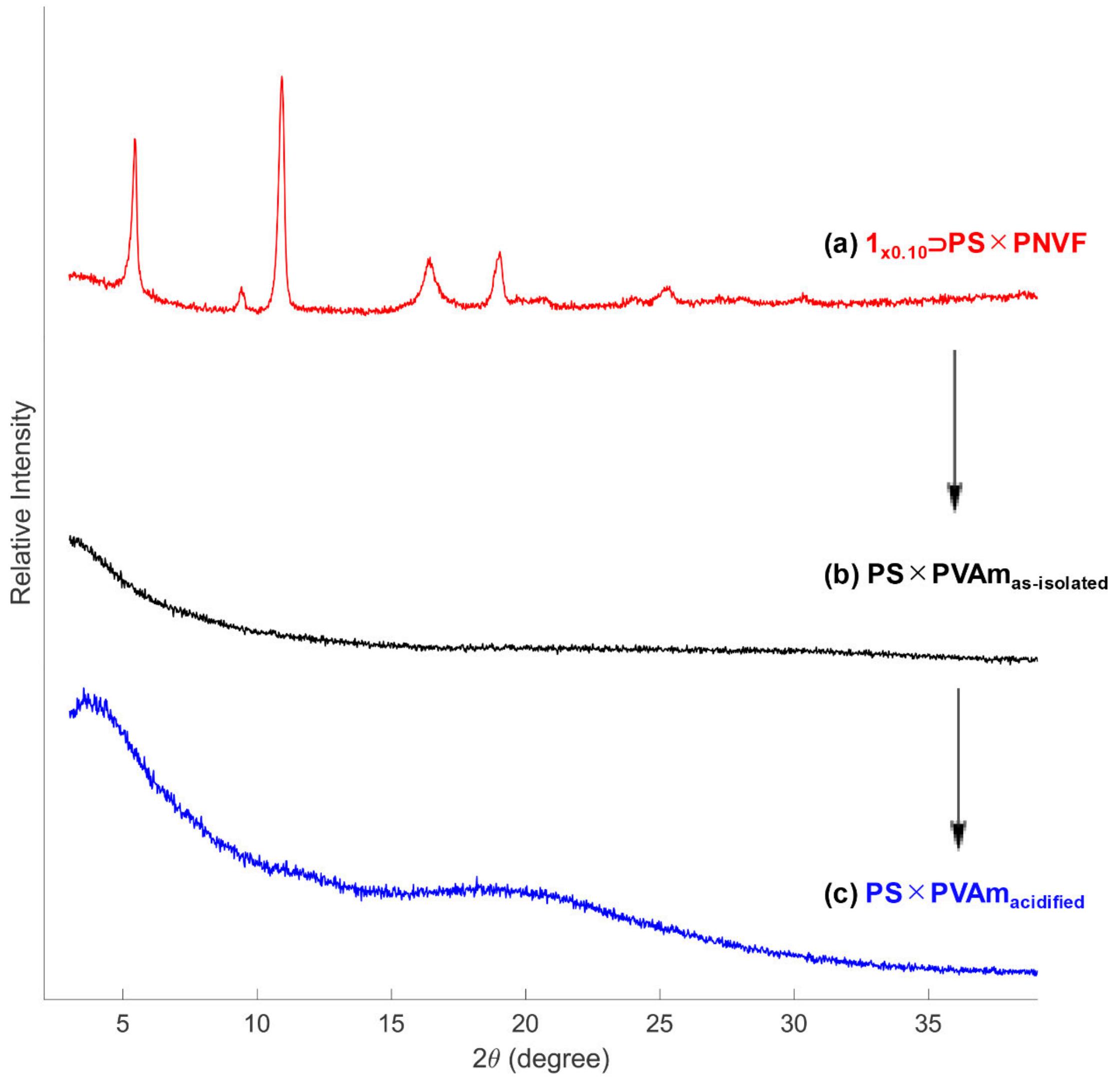

Supplementary Fig. 28. PXRD patterns of (a) $\mathbf{1_{x0.10}}$**⊃PS×PNVF**, (b) the **PS×PVAm** isolated from $\mathbf{1_{x0.10}}$, and (c) the same **PS×PVAm** after acidifying in conc. $HCl_{(aq)}$. Interestingly, a broad peak appears to have emerged at 2θ = ~4°, which is a lower angle than the lowest-angle reflection of the parent MOF, as well as in the ~20° region typically expected of amorphous polymers.

2. Supplementary Tables

Supplementary Table 1. Single-polymer composite synthesis by polymerization in **1**

Sample	Monomer loading	Initiator loading	Monomer conversion	Polymer loading	N_2 micropore volume (cm^3/g)	H_2O micropore volume (cm^3/g)
**1**	–	–	–	–	66.3 [d]	162.0 [e]
**1⊃PS**	10.0 wt%	BPO [a] 1.8 wt%	83.4%	8.3 wt%	2.9	133.9
**1⊃PMVK**	12.1 wt%	AIBN [b] 3.1 wt%	46.3%	5.6 wt%	7.5	126.5
**1⊃PAc**	13.7 wt%	AIBN [b] 3.6 wt%	48.9%	6.7 wt%	17.7	108.3
**1⊃PAAm**	13.3 wt%	APS [c] 1.6 wt%	77.3%	10.3 wt%	33.1	56.1
**1⊃PNVF**	13.4 wt%	AIBN [b] 1.0 wt%	99.5%	13.3 wt%	5.6	36.5

[a] BPO = benzoyl peroxide. Heat program: 120 °C 48 h, then 150 °C 48 h.
[b] AIBN = 2,2'-azobisisobutyronitrile. Heat program: 70 °C 48 h, then 100 °C 48 h.
[c] APS = ammonium persulfate. Heat program: 70 °C to 150 °C gradient, 96 h.
[d] Calculated through BET analysis on N_2 adsorption isotherm.
[e] Corresponds to the step height, *h*, of H_2O adsorption isotherm (Supplementary Fig. 6).

Supplementary Table 2. Molecular weight characterization of polymers extracted from **1**

Sample	Extracted polymer	M_n (g/mol) [a]	M_w (g/mol) [a]	*Đ*
**1⊃PS**	PS	570	1190	2.1
**1⊃PMVK**	PMVK	1470	2900	2.0
**1⊃PAc**	PAc	1250	3220	2.6
**1⊃PAAm**	PAm	2300	2940	1.3
**1⊃PNVF**	PNVF	1480	2700	1.8

[a] Determined by SEC calibrated with polystyrene standards.

Supplementary Table 3. Synthesis of **1⊃PS/PNVF** by simultaneous polymerization in **1**

Product	Monomer loading	AIBN [a] loading	Monomer conversion	Polymer loading	N_2 micropore volume (cm^3/g)	H_2O micropore volume (cm^3/g)
**1**	–	–	–	–	66.3 [b]	162.0 [c]
**1⊃PS /PNVF**	10.7 wt% **S** 7.1 wt% **NVF**	0.3 wt%	89.4% **S** 100% **NVF**	9.6 wt% **PS** 7.1 wt% **PNVF**	4.6	54.0

[a] AIBN = 2,2'-azobisisobutyronitrile. Heat program: 70 °C to 150 °C gradient, 96 h.
[b] Calculated through BET analysis on N_2 adsorption isotherm.
[c] Corresponds to the step height, *h*, of H_2O adsorption isotherm (Supplementary Fig. 6).

Supplementary Table 4. Synthesis of **1⊃PS/PNVF** by stepwise polymerization in **1**

Step	Product	Monomer loading	AIBN [a] loading	Monomer conversion	Polymer loading	N_2 micropore volume (cm^3/g)	H_2O micropore volume (cm^3/g)
0	**1**	–	–	–	–	66.3 [b]	162.0 [c]
1	**1⊃PS**	15.2 wt%	1.1 wt%	71.3%	11.8 wt%	15.5	134.8
2	**1⊃PS /PNVF**	7.7 wt%	1.0 wt%	98.3%	11.0 wt% **PS** 7.6 wt% **PNVF**	6.7	51.7

[a] AIBN = 2,2’-azobisisobutyronitrile. Heat program: 70 °C to 150 °C gradient, 96 h.
[b] Calculated through BET analysis on N_2 adsorption isotherm.
[c] Corresponds to the step height, *h*, of H_2O adsorption isotherm (Supplementary Fig. 6).

Supplementary Table 5. Synthesis and characterization of mixed-ligand mip/dvip MOFs

Product	Feed ratio (mip/dvip)	Incorporation ratio (mip/dvip)	*c*	N_2 micropore volume (cm^3/g) [a]	H_2O micropore volume (cm^3/g) [b]
$\mathbf{1_{x0.01}}$	0.970 / 0.030	0.986 / 0.014	0.01	68.2	143.5
$\mathbf{1_{x0.04}}$	0.940 / 0.060	0.964 / 0.036	0.04	50.1	127.3
$\mathbf{1_{x0.05}}$	0.910 / 0.090	0.950 / 0.050	0.05	59.2	135.8
$\mathbf{1_{x0.10}}$	0.820 / 0.180	0.903 / 0.097	0.10	55.9	166.5
$\mathbf{1_{x0.27}}$	0.700 / 0.300	0.731 / 0.269	0.27	51.2	32.6
$\mathbf{1_{x0.51}}$	0.400 / 0.600	0.495 / 0.505	0.51	36.3	–
$\mathbf{2_{x1.00}}$	0.000 / 1.000	0.000 / 1.000	1.00	17.4	–

[a] Calculated through BET analysis on N_2 adsorption isotherm.
[b] Determined as the step height, *h*, of H_2O adsorption isotherm (Supplementary Fig. 6).

Supplementary Table 6. Synthesis of **1⊃PS×PNVF** by stepwise polymerization in $\mathbf{1_{x0.10}}$

Step	Product	Monomer loading	BPO [a] loading	Monomer conversion	dvip conversion	N_2 micropore volume (cm^3/g) [b]	H_2O micropore volume (cm^3/g) [c]
0	$\mathbf{1_{x0.10}}$	–	–	–	–	55.9	166.5
1	$\mathbf{1_{x0.10}}$**⊃PS**	12.1 wt%	0.29 wt%	77.0%	34.0%	12.7	145.1
2	$\mathbf{1_{x0.10}}$**⊃PS ×PNVF**	10.8 wt%	0.26 wt%	100%	62.0%	7.5	20.2

[a] BPO = benzoyl peroxide. Heat program: 70 °C to 150 °C gradient, 96 h.
[b] Calculated through BET analysis on N_2 adsorption isotherm.
[c] Determined as the step height, *h*, of H_2O adsorption isotherm (Supplementary Fig. 6).

3. Supplementary Methods

Supplementary Method 1. Representative procedures for polymerization within 1.

Single-Monomer Polymerization of S. AIBN (5.9 mg) was dissolved in 5 mL of **S**. This solution was added to 0.52 g of **1** and sonicated for 30 min, followed by evaporation of the bulk **S** under 0.8 kPa vacuum at 30 °C. This yielded 0.60 g of **1⊃S**, whose **S** loading was determined by ^{1}H NMR. The composite was placed in a round-bottom flask equipped with a three-way stopcock and subjected to five vacuum-nitrogen cycles. The entire apparatus was then heated to 70 °C, followed by a linear temperature gradient program heating from 70-to-150 °C over the course of 96 h. It was then cooled, and the resulting **1⊃PS** was subjected to further analysis.

Single-Monomer Polymerization of NVF. AIBN (3.6 mg) and **NVF** (45 µL) were dissolved in 2 mL of acetone. This solution was added to 0.31 g of **1** and sonicated for 30 min, followed by evaporation of acetone at 30 °C under an 11 kPa vacuum. The resulting powder was redispersed in acetone and processed once more under the same conditions for improved homogeneity, yielding 0.36 g of **1⊃NVF**. The NVF loading was determined by ^{1}H NMR. The composite was placed in a round-bottom flask equipped with a three-way stopcock and subjected to five vacuum-nitrogen cycles. The entire apparatus was then heated to 70 °C, followed by a linear temperature gradient program heating from 70-to-150 °C over the course of 96 h. It was then cooled, and the resulting **1⊃PNVF** was subjected to further analysis.

Two-Monomer Simultaneous Polymerization of S and NVF. AIBN (3.0 mg) and **NVF** (150 µL) were dissolved in 2 mL of **S**. The resulting solution was added to 1.0 g of **1** and sonicated for 30 min. The bulk **S** was then removed by evaporation under 0.8 kPa vacuum at 30 °C. The powder was re-dispersed in acetone and dried again at 30 °C under an 11 kPa vacuum to enhance homogeneity, producing 1.17 g of **1⊃S/NVF**. The loadings of **S** and **NVF** were quantified by ^{1}H NMR. The composite was placed in a round-bottom flask equipped with a three-way stopcock and subjected to five vacuum-nitrogen cycles. The entire apparatus was then heated to 70 °C, followed by a linear temperature gradient program heating from 70-to-150 °C over the course of 96 h. It was then cooled, and the resulting **1⊃PS/PNVF** was subjected to further analysis.

Supplementary Method 2. Le Bail analysis.

PXRD data for Le Bail analysis was gathered from capillary samples in transmission (Debye-Scherrer) mode using Cu Kα radiation, a scan rate of 0.256 degrees per minute, sampling resolution of 0.0128 degrees, and rotation rate of 100 rpm. Capillary samples were activated at 150 °C under vacuum to desorb any low-MW guests, and flame sealed prior to measurement. Le Bail fitting of this PXRD data was carried out using the Jana2020 software package,[5] giving both cell parameters for DFT structure refinement (Supplementary Method 3) and structure factors for MEM analysis (Supplementary Method 4). Cell parameters derived from these analyses are summarized in Supplementary Method Table 1.

Supplementary Method 3. Density functional theory (DFT) calculations.

Periodic DFT-optimized models of gate-open and gate-closed forms of **1** were obtained through geometry optimization using the PBEsol functional[6,7] with Grimme's D3 damped dispersion terms[8] as implemented in the CP2K software package.[9] The DZVP-MOLOPT basis set was used for Cu atoms, and the TZVP-MOLOPT basis set for C, H, and O atoms. All calculations used a plane wave cutoff energy of 600 Ry, and core electron pseudopotentials for

all atoms as formulated by Geodecker *et al.*[10] Starting models were derived from the reported hydrated (gate-open) and dehydrated (gate-closed)[1] with ether groups (-OEt) substituted for methyl groups ($-CH_3$) and cell parameters scaled to those experimentally obtained from Le Bail fitting of XRD data (Supplementary Method Table 1).

As these DFT calculations are incompatible with the partial crystallographic occupancy of sites by adsorbed water in the gate-open form (as seen in STAM-17-OEt),[1] one of three possible sites was chosen for each disordered water molecule in the unit cell, resulting in a final water content of 10 molecules per unit cell, which is in line with the number suggested by TG curves and vapor sorption experiments (Fig. 1, Supplementary Fig. 7).

Electrostatic charges for use in Monte Carlo simulations were determined using the REPEAT method,[11] as implemented in CP2K (Supplementary Method Table 2). As the deletion of water molecules from the model for gate-open form resulted in a residual positive charge on the framework, the charges of negative atoms were scaled up to compensate and return the overall charge to 0. Charges on the gate-closed form were neutral overall and could be used as-is. These charged, guest-free models were then used as adsorbents for Monte Carlo simulations in RASPA (see Method).

Supplementary Method Table 1. Unit cell parameters as determined by Le Bail analysis of PXRD data, and used in the optimization of structural models by periodic DFT.

Model	*a* (Å)	*b* (Å)	*c* (Å)	*α* (°)	*β* (°)	*γ* (°)
Gate-open (GO)	18.515	18.515	6.896	90	90	120
Gate-closed (GC)	33.040	33.040	5.203	90	90	120

Supplementary Method Table 2. Averaged Lennard-Jones parameters and REPEAT charges by atom type. An illustration of one stoichiometric unit of **1** is provided as legend.

Legend	Atom type	*ε*/kB (K)	*σ* (Å)	Avg. *q* (e) (gate-open)	Avg. *q* (e) (gate-closed)
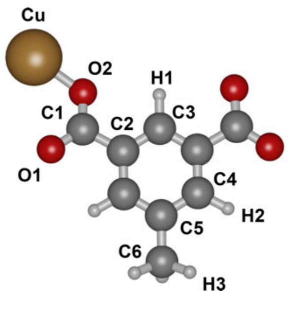	Cu	2.518	3.114	1.418	0.973
	O1	109.702	3.118	-0.647	-0.550
	O2	109.702	3.118	-0.638	-0.513
	C1	48.812	3.434	0.542	0.537
	C2	48.812	3.434	0.110	0.199
	C3	48.812	3.434	-0.031	-0.421
	C4	48.812	3.434	-0.587	-0.389
	C5	48.812	3.434	0.574	0.459
	C6	48.812	3.434	-0.695	-0.632
	H1	5.032	2.576	0.069	0.226
	H2	5.032	2.576	0.287	0.169
	H3	5.032	2.576	0.177	0.163

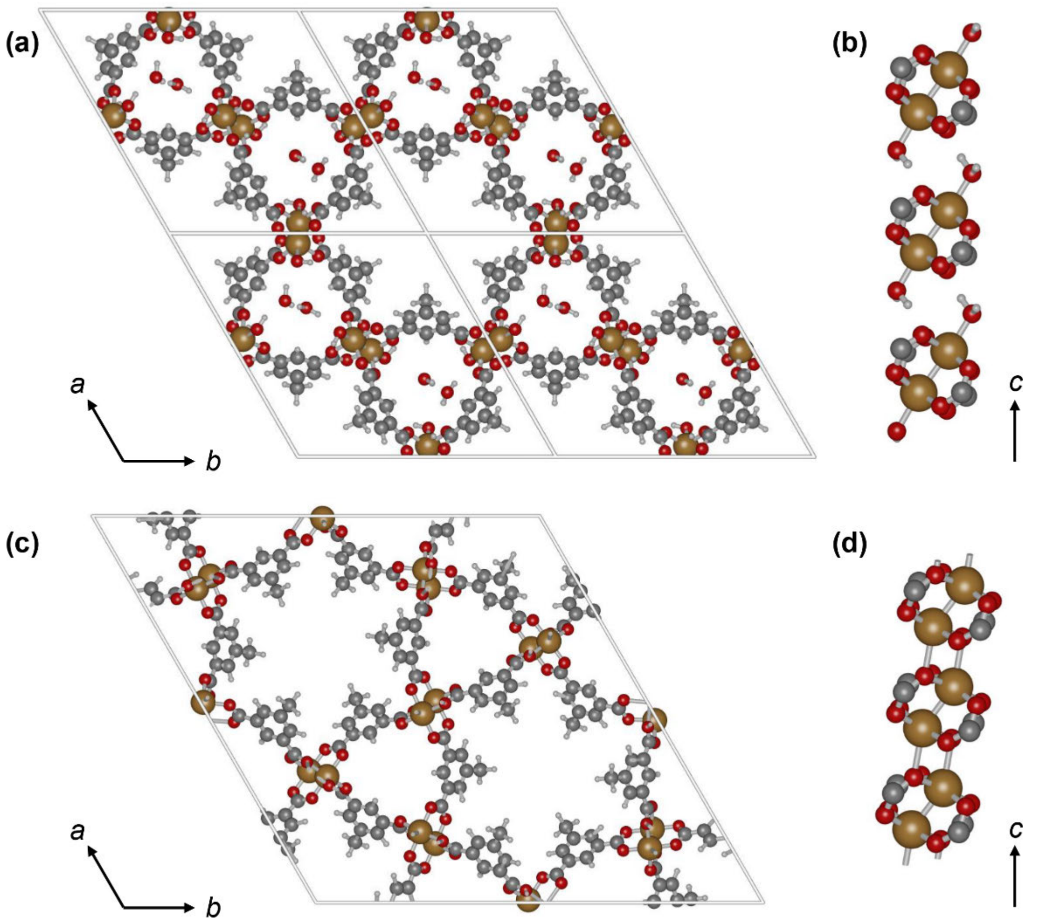

Supplementary Method Fig. 1. DFT-optimized structures of **1** in (a, b) gate-open hydrated form and (c, d) gate-closed form. Views along and orthogonal to the (0 0 1) axes are provided to illustrate the pore structure and paddlewheel coordination state respectively. The white parallelograms illustrate the different sizes of the respective unit cells. Discrete H_2O were used only for DFT geometry optimization of the gate-open form and removed from the model for GCMC simulations.

Supplementary Method 4. Maximum Entropy Method (MEM) analysis.

MEM analyses were carried out using the Dysnomia software package,[12] using the observed structure factors and their estimated standard uncertainties determined from profile fits (Supplementary Method 2) as exported from Jana2020.[5] By importing DFT-optimized structural models to Jana2020, phase information from the MOF template was applied to assist in converging the electron density map by MEM.

MEM analyses were converged from a flat prior with constraints on the second- and fourth-order central moments. As estimated standard uncertainties are often over- or under-estimated from pattern fitting and can depend upon the software used to determine them, Dysnomia uses a user-defined empirical adjustment factor ‘E’ to scale them up or down. In this work, E was varied over multiple MEM analyses and final results were taken to be those where the lowest R-factors were obtained while still converging to satisfy the maximum entropy constraint within 200,000 iterations of the L-BFGS algorithm.

Supplementary Method 5. Synthesis of dvipH_2.

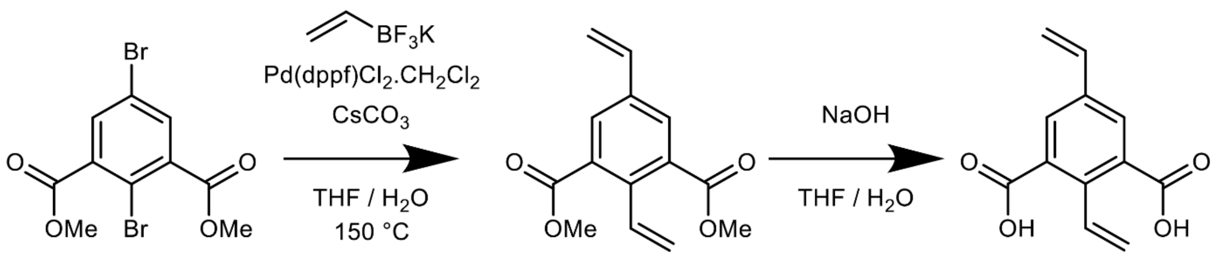

Dimethyl 2,5-dibromoisophthalate was obtained by oxidation of 2,5-dibromo-*m*-xylene to 2,5-dibromoisophthalic acid, followed by esterification to dimethyl 2,5-dibromoisophthalate and purification by flash chromatography as reported elsewhere.[13]

A 20 mL microwave reaction vial (Biotage Initiator+) with magnetic stir bar was charged with dimethyl 2,5-dibromoisophthalate (0.7 g, 2.0 mmol), potassium vinyltrifluoroborate (682 mg, 5.1 mmol), cesium carbonate (3988 mg, 12.2 mmol), and $Pd(dppf)Cl_2 \cdot CH_2Cl_2$ (84 mg, 0.1 mmol, dppf = bis(diphenylphosphino)ferrocene) in 18mL tetrahydrofuran and 2 mL H_2O. It was sealed with a PTFE-lined cap and subjected to pressurized microwave heating at 150 °C for 1 hour. The organic phase was then taken, concentrated by rotary evaporator and subjected to gradient flash chromatography (hexane/ethyl acetate, v/v, 0-100%). Unfortunately, the target dimethyl 2,5-divinylisophthalate could not be chromatographically separated from the mono-vinylated side product at this stage so both were subjected to the next step.

The fraction containing dimethyl 2,5-divinylisophthalate was dissolved in 40 mL THF and added to 20 mL 1 M $NaOH_{(aq)}$ in a 100 mL round-bottom flask equipped with a magnetic stir bar. The flask was stirred for 48 hour at room temperature, then the aqueous phase was extracted and washed with 3 × 20 mL of dichloromethane. It was then acidified to pH ~1 by conc. $HCl_{(aq)}$ upon addition of which a white precipitate formed. This was then extracted by 3 × 20 mL of ethyl acetate, then the combined dried organic fractions recrystallized from boiling $CHCl_3$ to yield 2,5-divinylisophthalic acid (dvipH_2) (58.8 mg, 0.027 mmol, 13% yield).

^{1}H NMR (500 MHz, $CDCl_3$): δ (ppm) 8.02 (s, 2H), 7.38 (dd, J = 17.6 and 11.3 Hz, 1H), 6.73 (dd, J = 17.6 and 11.1 Hz, 1H), 5.88 (d, J = 17.6 Hz, 1H), 5.4-5.45 (m, 2H), 5.28 (d, J = 17.6 Hz, 1H); ^{13}C NMR (125 MHz): δ (ppm) 169.1, 136.5, 135.2, 135.0, 133.9, 128.5, 118.9, 117.1; HRMS (ESI-negative): calcd. for $C_{12}H_9O_4$ $[M-H]^-$: m/z = 217.05063; found 217.05240.

4. Supplementary References

1. McHugh, L. N. *et al.* Hydrolytic stability in hemilabile metal–organic frameworks. *Nat. Chem.* **10**, 1096–1102 (2018).
2. Dalby, O. P. L., Abbott, S., Matubayasi, N. & Shimizu, S. Cooperative sorption on heterogeneous surfaces. *Langmuir* **38**, 13084–13092 (2022).
3. Law, R. V., Sherrington, D. C., Snape, C. E., Ando, I. & Kurosu, H. Solid-state ^{13}C MAS NMR studies of hyper-cross-linked polystyrene resins. *Macromolecules* **29**, 6284–6293 (1996).
4. Uemura, T. *et al.* Mixing of immiscible polymers using nanoporous coordination templates. *Nat Commun* **6**, 7473 (2015).
5. Petříček, V., Palatinus, L., Plášil, J. & Dušek, M. Jana2020 – a new version of the crystallographic computing system Jana. *Z. für Krist. - Cryst. Mater.* **238**, 271–282 (2023).
6. Constantin, L. A., Perdew, J. P. & Pitarke, J. M. Exchange-correlation hole of a generalized gradient approximation for solids and surfaces. *Phys. Rev. B* **79**, 075126 (2009).
7. Perdew, J. P. *et al.* Restoring the density-gradient expansion for exchange in solids and surfaces. *Phys. Rev. Lett.* **100**, 136406 (2008).
8. Grimme, S., Antony, J., Ehrlich, S. & Krieg, H. A consistent and accurate *ab initio* parametrization of density functional dispersion correction (DFT-D) for the 94 elements H-Pu. *J. Chem. Phys.* **132**, 154104 (2010).
9. Hutter, J., Iannuzzi, M., Schiffmann, F. & VandeVondele, J. cp2k: atomistic simulations of condensed matter systems. *Wiley Interdiscip. Rev.: Comput. Mol. Sci.* **4**, 15–25 (2014).
10. Goedecker, S., Teter, M. & Hutter, J. Separable dual-space Gaussian pseudopotentials. *Phys. Rev. B* **54**, 1703–1710 (1995).
11. Campañá, C., Mussard, B. & Woo, T. K. Electrostatic potential derived atomic charges for periodic systems using a modified error functional. *J. Chem. Theory Comput.* **5**, 2866–2878 (2009).
12. Momma, K., Ikeda, T., Belik, A. A. & Izumi, F. Dysnomia, a computer program for maximum-entropy method (MEM) analysis and its performance in the MEM-based pattern fitting. *Powder Diffr.* **28**, 184–193 (2013).
13. Surampudi, S. K., Nagarjuna, G., Okamoto, D., Chaudhuri, P. D. & Venkataraman, D. Apical functionalization of chiral heterohelicenes. *J. Org. Chem.* **77**, 2074–2079 (2012).
